# Experimental and computational investigation on the charge storage performance of a novel Al_2_O_3_-reduced graphene oxide hybrid electrode

**DOI:** 10.1038/s41598-022-23574-2

**Published:** 2023-03-31

**Authors:** Satyajit Ratha, Surjit Sahoo, Pratap Mane, Balaram Polai, Bijoy Sathpathy, Brahmananda Chakraborty, Saroj Kumar Nayak

**Affiliations:** 1 School of Basic Sciences, Indian Institute of Technology Bhubaneswar, Argul, Khordha, 752050 India; 2 Seismology Division, Bhabha Atomic Research Centre, Trombay, Mumbai, 400085 India; 3 High Pressure and Synchroton Radiation Physics Division, Bhabha Atomic Research Centre, Trombay, Mumbai, 400085 India; 4 Homi Bhabha National Institute, Mumbai, 400094 India

**Keywords:** Materials science, Materials for energy and catalysis, Electrochemistry

## Abstract

The advancements in electrochemical capacitors have noticed a remarkable enhancement in the performance for smart electronic device applications, which has led to the invention of novel and low-cost electroactive materials. Herein, we synthesized nanostructured Al_2_O_3_ and Al_2_O_3_-reduced graphene oxide (Al_2_O_3_-rGO) hybrid through hydrothermal and post-hydrothermal calcination processes. The synthesized materials were subject to standard characterisation processes to verify their morphological and structural details. The electrochemical performances of nanostructured Al_2_O_3_ and Al_2_O_3_- rGO hybrid were evaluated through computational and experimental analyses. Due to the superior electrical conductivity of reduced graphene oxide and the synergistic effect of both EDLC and pseudocapacitive behaviour, the Al_2_O_3_- rGO hybrid shows much improved electrochemical performance (~ 15-fold) as compared to bare Al_2_O_3_. Further, a symmetric supercapacitor device (SSD) was designed using the Al_2_O_3_- rGO hybrid electrodes, and detailed electrochemical performance was evaluated. The fabricated Al_2_O_3_- rGO hybrid-based SSD showed 98.56% capacity retention when subjected to ~ 10,000 charge–discharge cycles. Both the systems (Al_2_O_3_ and its rGO hybrid) have been analysed extensively with the help of Density Functional Theory simulation technique to provide detailed structural and electronic properties. With the introduction of reduced graphene oxide, the available electronic states near the Fermi level are greatly enhanced, imparting a significant increment in the conductivity of the hybrid system. The lower diffusion energy barrier for electrolyte ions and higher quantum capacitance for the hybrid structure compared to pristine Al_2_O_3_ justify improvement in charge storage performance for the hybrid structure, supporting our experimental findings.

## Introduction

The conventional resources utilized to provide the world's energy requirements, such as coal, oil, natural gas, and petroleum products, are facing rapid exhaustion. On the other hand, the combustion of these fossil fuels creates severe environmental issues. Therefore, it is crucial to find alternative energy resources that are sustainable, clean, and abundant. In the current scenario, electrochemical energy storage devices are crucial in overcoming fossil fuel depletion and global warming^1–3^. Among miscellaneous electrical energy storage devices, rechargeable batteries and electrochemical capacitors have drawn significant interest, due to their widespread application in industries and our daily lives, for the last few years, because of their impressive electrochemical profile, characterised by excellent cyclic stability, high rate of charge/discharge, balanced specific energy, and power density values^4,5^. Supercapacitors (both electric double layer capacitors (EDLCs) and pseudocapacitors) have the advantage of releasing energy in a shorter time by using their fast surface or near surface electrochemical reactions (surface adsorption/diffusion processes)^4,6^. In the case of both EDLC and pseudocapacitive types of supercapacitors, the most frequently used materials (for electrode fabrication), for academia and industries, including both the carbon-based (for EDLC) and transition-metal-based compounds (for pseudocapacitor)^7,8^. Among various transition metal compounds, transition metal oxides usually have lower electrical conductivity than carbon-based materials. Likewise, in the case of carbon-based materials, we often observe uneven morphologies and wide pore size distributions. Compared to materials mentioned earlier, oxides of transition group metals are a prominent group of materials for supercapacitors^6,9^. Metal oxides have unique chemical, physical and electrochemical properties, such as mechanical, thermal stability, and high capacity. They can be easily synthesized and are quite cheaper than metal hydroxides, carbon-based active materials, and conducting polymers^10,11^.

Aluminum (Al) has the highest abundance (amongst metals) in the earth's crust and is used in various research fields or day-to-day lives due to its low cost and lightweight nature. Previously, Huang et al*.* used commercial Al foil as the current collector for supercapacitor application. The laser-treated Al current collector shows better electrochemical performance (higher capacitance and cyclic stability) than the pristine-Al current collector due to lower internal resistances^12^. Aluminum oxide (Al_2_O_3_) has many distinctive and attractive behaviours, such as good thermal conductivity, inertness to most acids and alkalis, high mechanical strength, high adsorption capacity, good thermal stability, non-toxicity, and cost-effectiveness^13–15^. These fascinating properties compel Al_2_O_3_ nanostructures to be a potential material for catalysis, sensors, batteries, and supercapacitors. To use Al_2_O_3_ nanostructures as electrode material for supercapacitors, Dia et al.^16^ synthesized an Al_2_O_3_-ZnO composite using the solvent precipitation method for supercapacitor application. Even though the electrochemical behaviour of electroactive material Al_2_O_3_ nanostructures has been found suitable for energy storage applications, electrochemical performance in aspects of gravimetric capacitance, specific energy, and specific power, still needs further improvement. The combination of an EDLC material with a pseudocapacitive material can impart a balance between energy density and power density. The EDLC material typical has high power density with low energy density, while a pseudocapacitive material has high energy density with moderate power density. When integrated into composite or hybrids, EDLC and pseudocapacitive materials will provide significant improvement which is otherwise could not be obtained individually^17,18^. Reduced graphene oxide can enhance the electrical conductivity, mechanical stability, and surface area of electrochemically active metal oxides for supercapacitor application, the use of reduced graphene oxide hybrid is one of the most effective routes^19,20^.

In the current work, we describe a low-cost, environmentally benign, and easily scalable method to prepare an Al_2_O_3_-reduced graphene oxide hybrid for supercapacitor application. We performed detailed electrochemical characterisation of pristine Al_2_O_3_ and Al_2_O_3_-reduced graphene oxide hybrid, along with computational analyses. Interestingly, the Al_2_O_3_-reduced graphene oxide hybrid shows higher storage performance (~ 15-fold higher) than pristine Al_2_O_3_, which can be attributed to the synergistic effect of both EDLC and pseudocapacitive behaviour. To support our experimental data as well as to get theoretical insights regarding interactions between Al_2_O_3_ and reduced graphene oxide, we have done Density Functional Theory simulations for Al_2_O_3_ and Al_2_O_3_-reduced graphene oxide hybrid.

## Experimental section

### Materials

Aluminium nitrate nonahydrate [Al(NO_3_)_3_.9H_2_O], glucose anhydrous [C_6_H_12_O_6_], ammonia (NH_3_.H_2_O), and sodium sulfate (Na_2_SO_4_) were purchased from Merck, India. Activated carbon (AC) and PVDF were bought from Alfa Aesar (India). The commercial grade stainless steel (grade-304, thickness: 1.0 mm) obtained from Amazon (India) was used as the substrate for the electrochemical study. All stainless-steel substrates were thoroughly cleaned with ethanol and DI water many times by ultra-sonication process and polished using sandpapers. Then they were dried in an electric hot-air oven at 60 ℃ for one hour and afterward used as the substrate for electroactive material coating.

### Preparation of Al_2_O_3_ nanostructures

Nanostructured Al_2_O_3_ was prepared through a standard hydrothermal reaction protocol, followed by a high-temperature calcination step. In a typical synthesis protocol, 3.75 g of Al(NO_3_)_3_.9H_2_O and 2.5 g of C_6_H_12_O_6_ were mixed in 60 mL of Millipore water using an ultrasonication mixer. Afterward, 10 mL of NH_3_·H_2_O solution was added to the above precursor solution, and the final mixture solution was subject to ultra-sonication for ~ 1.5 h. The mixture was then carefully transferred to a Teflon-lined stainless steel autoclave of 100 mL capacity and was kept for 24 h at 200 °C in a hot air oven. Precipitates were collected once the hydrothermal reaction was complete, repeatedly washed via several centrifugation steps (using absolute ethanol), and dried at 70 °C overnight. After drying, the as-synthesized sample was thermally treated at 1100 ºC for five hours to obtain the product.

### Synthesis of Al_2_O_3_-reduced graphene oxide hybrid

**Figure 1:**
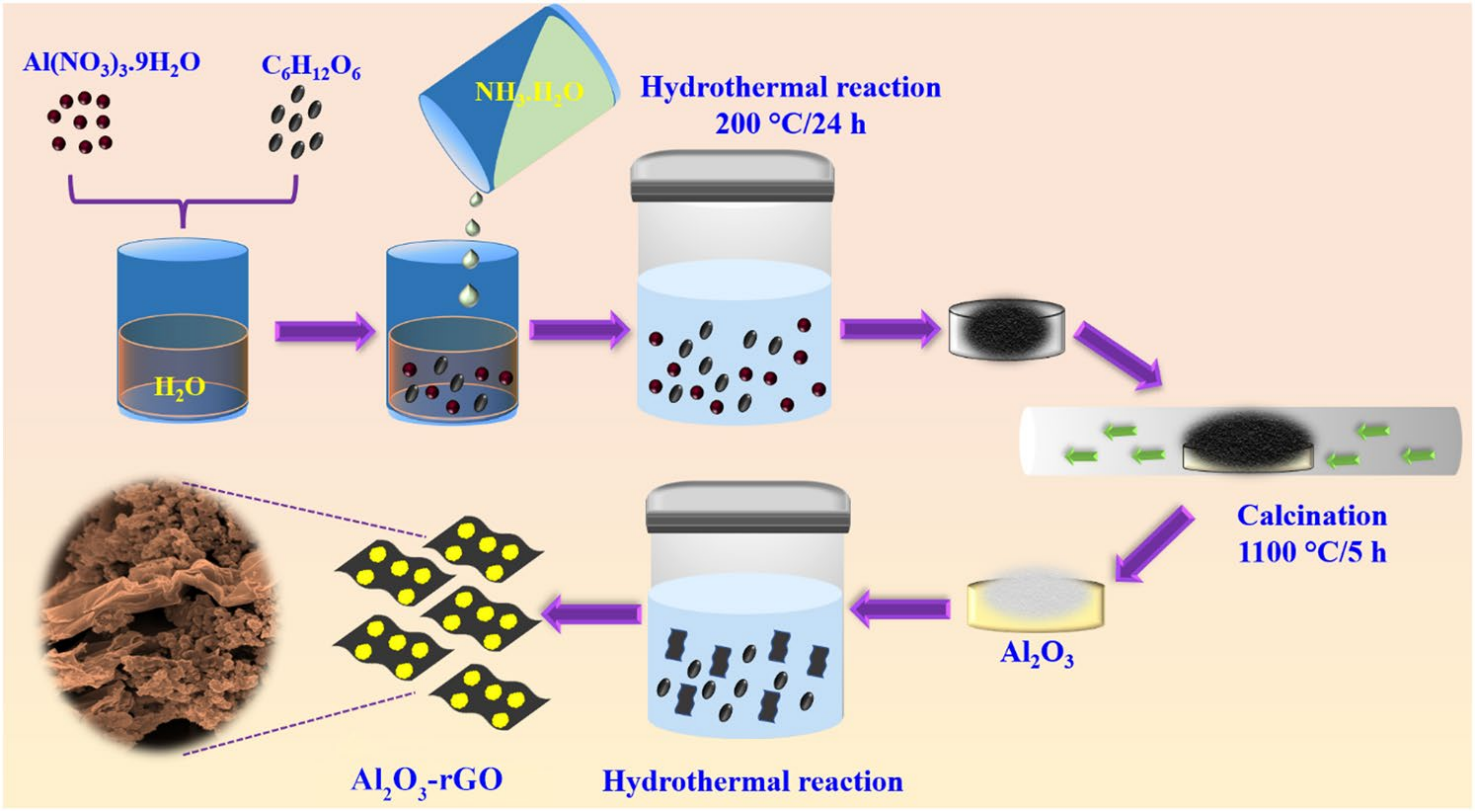
Schematic illustration for preparation of nanostructured Al_2_O_3_ and Al_2_O_3_-reduced graphene oxide hybrid.

The commercial grade graphene oxide (GO) powder (purity > 99%) was purchased from ULTRANANOTECH (India). In a typical approach, GO was dispersed in 50 mL of de-ionized water to form a homogeneous GO dispersion by ultra-sonication for about two hours. Then the prepared (aforementioned) Al_2_O_3_ powder was added to the GO dispersion solution, which was subsequently kept under ultra-sonication for one hour. Finally, the mixture solution was transferred to a 100 mL autoclave (Teflon lined stainless steel) and kept in a hot air oven at 180 °C for 15 h. The precipitated Al_2_O_3_-reduced graphene oxide hybrid was centrifuged in absolute ethanol and kept inside a hot air oven to dry at 60 °C overnight. The details schematic representation of the synthesis process for both the Al_2_O_3_ and Al_2_O_3_-reduced graphene oxide hybrid has been provided in Fig. 1.

### Physical characterization

The XRD analysis of the Al_2_O_3_ and Al_2_O_3_-reduced graphene oxide hybrid was obtained using a Bruker D8 Advance X-ray diffractometer instrument at 40 kV and 40 mA with Cu-Kα radiation. The surface morphology of the as-prepared Al_2_O_3_ and Al_2_O_3_-reduced graphene oxide hybrid nanostructure was analyzed using field-emission scanning electron microscopy (FE-SEM; MERLIN Compact with Gemini-I electron column) under different magnifications and energy dispersive X-ray spectroscopy (EDS) was carried out on Oxford instruments. The BET surface area analysis was done using Quanta chrome (Model: Quadrasorbevo 3), USA. The oxidation states of the constituent elements in the Al_2_O_3_-reduced graphene oxide hybrid were confirmed with the help of X-ray Photoelectron Spectroscopy (ULVAC PHI (Physical Electronics), USA (Model: PHI 5000 VERSA PROBE III)). High-Resolution Transmission Electron Microscopy (HR-TEM- JEOL JEM 2100 PLUS) analyses were performed for the Al_2_O_3_-reduced graphene oxide hybrid to find morphological, crystallographic, and compositional information.

### Electrochemical characterization

As reported previously, the fabrication of the working electrode was done with the help of the slurry coating method on stainless-steel substrates^21^. The process was carried out by preparing a slurry, where the electroactive material (Al_2_O_3_ and Al_2_O_3_-reduced graphene oxide hybrid), AC, and PVDF (mixture weight ratio 85:10:5) were mixed and ground together with a viable proportion of N-methyl pyrrolidone (NMP) as the dispersing medium, using an agate-mortar. In the next step, the prepared slurry was coated on the surface of a pre-cleaned stainless-steel substrate of an area of 1 × 1 cm^2^ and was kept for drying in an electric oven. The mass loading in the case of both Al_2_O_3_ and Al_2_O_3_-reduced graphene oxide hybrid electrodes was ~ 1 mg each, which was calculated by noting the difference in the weights of the bare and the coated stainless-steel electrode, with the help of a precision weighing balance (AUW-220D, SHIMADZU).

For three electrode measurements, Al_2_O_3_ and Al_2_O_3_-reduced graphene oxide hybrid coated stainless steel served as the working electrode, while Ag/AgCl and platinum were used as the reference and counter electrodes, respectively. The Al_2_O_3_-reduced graphene oxide hybrid based 2-electrode symmetric device (SSD) was assembled by using a Whatman filter paper, which electrically isolates the two Al_2_O_3_-reduced graphene oxide hybrid electrodes. The electrochemical performances of the Al_2_O_3_ electrode, Al_2_O_3_-reduced graphene oxide hybrid electrode, and Al_2_O_3_-reduced graphene oxide hybrid symmetric cell device (SSD) were evaluated through potentiodynamic cyclic voltammetry (CV), galvanostatic charge–discharge (CD), and electrochemical impedance spectroscopy (EIS) techniques, using a multichannel Biologic electrochemical workstation (model no: VSP-300), by taking 1 M aqueous solution of Na_2_SO_4_ as the electrolyte. The gravimetric capacitance (*C*_*sp*_), specific energy (Es), and specific power (Ps) of the Al_2_O_3_ electrode, Al_2_O_3_-reduced graphene oxide hybrid electrode, and Al_2_O_3_-reduced graphene oxide hybrid SSD were evaluated via the following Eqs.^21^:

1 $$C_{sp} = \left[ {\left( {\smallint I\,\,dV} \right)/2\left( {s \times \Delta V \times m} \right)} \right]$$

2 $$C_{sp} = \left[ {\left( {I \times \Delta t} \right)/\left( {\Delta V \times m} \right)} \right]$$

3 $$Es = \left( {I \times \Delta t \times \Delta V} \right)/\left( {7.2 \times m} \right)$$

4 $$Ps = \left( {3.6 \times Es} \right)/\Delta t$$

Here, “*C*_*sp*_” denotes the gravimetric capacitance (F g^−1^), “*I*” represents the applied current (A), “*∆V*” is the electrochemically stable potential range, “*s*” is the sweep rate (mV s^−1^), “*∆t*” is the discharge duration (s), and “*m*” is the mass-loading in gram.

### Computational details

For electronic structure computations, we have implemented plane wave DFT package VASP(VIENNA ab initio simulation package) code^22,23^. The pseudopotentials employed for the present system were based on the projector-augmented wave (PAW) functional^24^. The GGA exchange-correlational functional was used to undertake ion–electron interactions^25,26^. For optimization and electronic calculations, the convergence criteria were set as 0.01 eV/Å and 10^–5^ eV for force and energy, respectively. For a plane-wave basis, kinetic energy cut-off was taken at 500 eV. The Brillouin zones were sampled using a K points mesh with the Monkhorst–Pack grid of 5 × 5 × 3 and 5 × 5 × 1 for bulk and surface optimization while a 7 × 7 × 3 and 7 × 7 × 1 for electronic energy calculations, respectively^27^.

## Results and discussion

**Figure 2:**
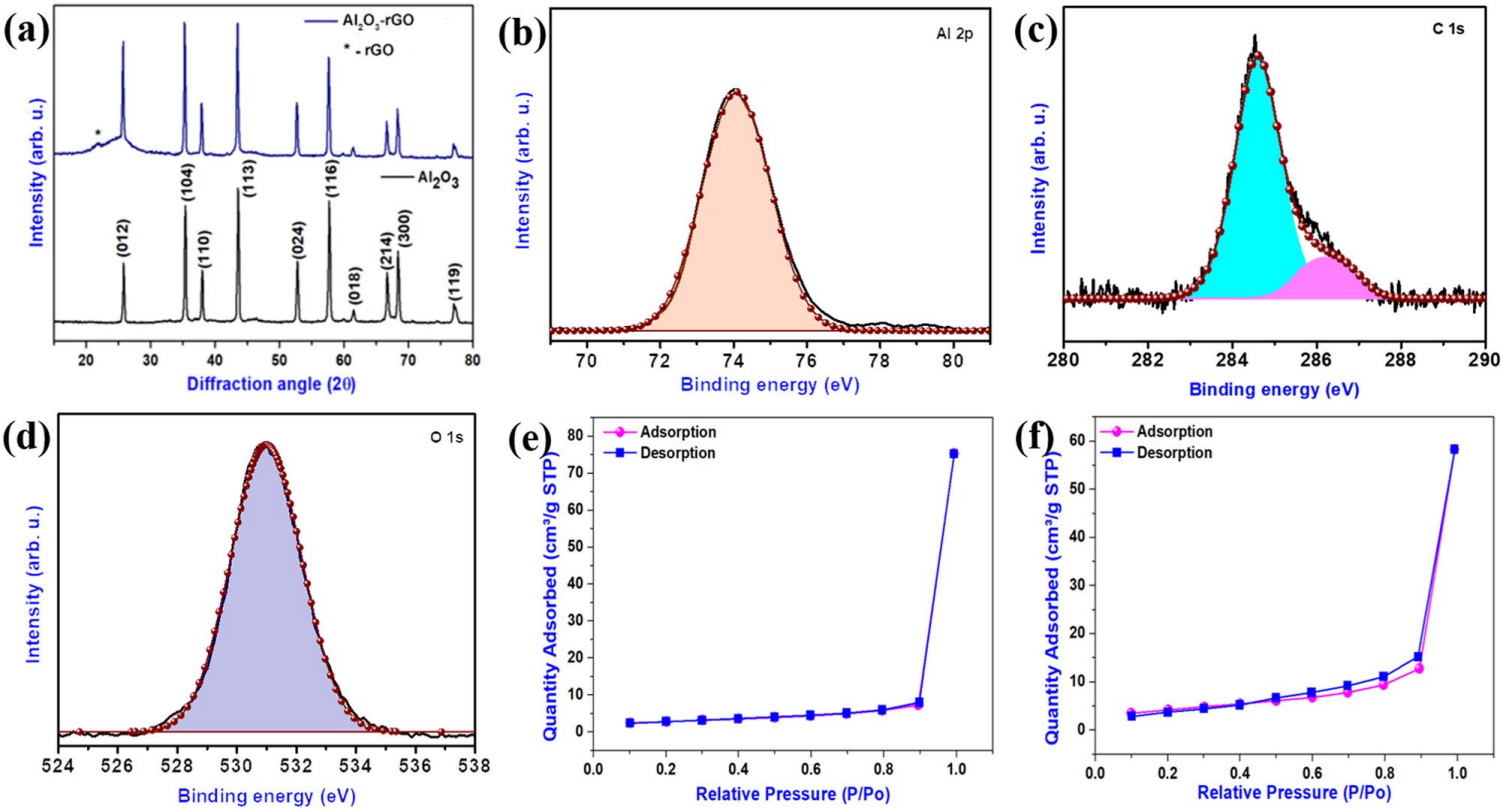
(**a**) Comparative XRD pattern of nanostructured Al_2_O_3_ and Al_2_O_3_-reduced graphene oxide hybrid. High-resolution XPS spectra for (**b**) Al 2p, (**c**) O 1 s, and (**d**) C 1 s in Al_2_O_3_-reduced graphene oxide hybrid. The Brunauer–Emmett–Teller (BET) analysis of pristine Al_2_O_3_ and Al_2_O_3_-reduced graphene oxide hybrid. The BET isotherms of (**e**) bare Al_2_O_3_ and (**f**) Al_2_O_3_-reduced graphene oxide hybrid.

Figure 2a represents the Al_2_O_3_ and Al_2_O_3_-reduced graphene oxide hybrid XRD comparison chart. The X-ray diffraction pattern for reduced graphene oxide has been provided in Fig. S1. In the Al_2_O_3_ XRD pattern, the peaks at 25.77°, 35.36°, 38.06°, 43.65°, 52.75°, 57.64°, 61.54°, 66.73°, 68.33°, and 77.23°, are ascribed to (012), (104), (110), (113), (024), (116), (018), (214), (300), and (119) reflections of rhombohedral Al_2_O_3_ (PDF:43-1484), respectively, demonstrating the successful formation of the Al_2_O_3_ nanostructure^28^. In the case of the Al_2_O_3_-reduced graphene oxide hybrid, all the peaks are well matched with rhombohedral Al_2_O_3_ with an additional broad peak at 2θ = 23°. The peak at 2θ = 23° indicates the (002) plane of reduced graphene oxide sheets via the hydrothermal reduction method. The XRD pattern suggests the formation of high-purity and high-crystalline Al_2_O_3_ and Al_2_O_3_-reduced graphene oxide hybrid. X-ray photoelectron spectroscopy (XPS) was employed to examine the bonding situation and electronic states in the Al_2_O_3_-reduced graphene oxide hybrid^29^. Figure 2b represents the high-resolution XPS spectrum for Al 2p, showing a peak at 74.02 eV, which is ascribed to Al^3+^ in Al_2_O_3_. The peak observed (in Fig. 2c) at 531.5 eV is attributed to O 1 s, which is associated with bonds of different types, due to the presence of additional functional groups such as C=O (at ~ 531 eV), (CO*)OH (at ~ 532 eV), -OH (at ~ 533 eV)^30^. Likewise, the C 1 s high-resolution XPS spectrum (in Fig. 2d) is deconvoluted into two peaks corresponding to C–C (284.6 eV) and C–OH (286.5 eV). All these XPS spectra are in good agreement with previously reported work^31,32^. The BET isotherms of bare Al_2_O_3_ and Al_2_O_3_-reduced graphene oxide hybrid are illustrated in Fig. 2e,f. The BET isotherms exhibit the typical type (IV) adsorption–desorption isotherms, which is characteristic of mesoporous materials^33,34^. The BET surface areas of Al_2_O_3_-reduced graphene oxide hybrid and pristine Al_2_O_3_, respectively, are about 15.100 m^2^/g and 9.724 m^2^/g. The BET results indicate that the surface area of the Al_2_O_3_-reduced graphene oxide hybrid is 1.5 times higher than that of pristine Al_2_O_3_.

**Figure 3:**
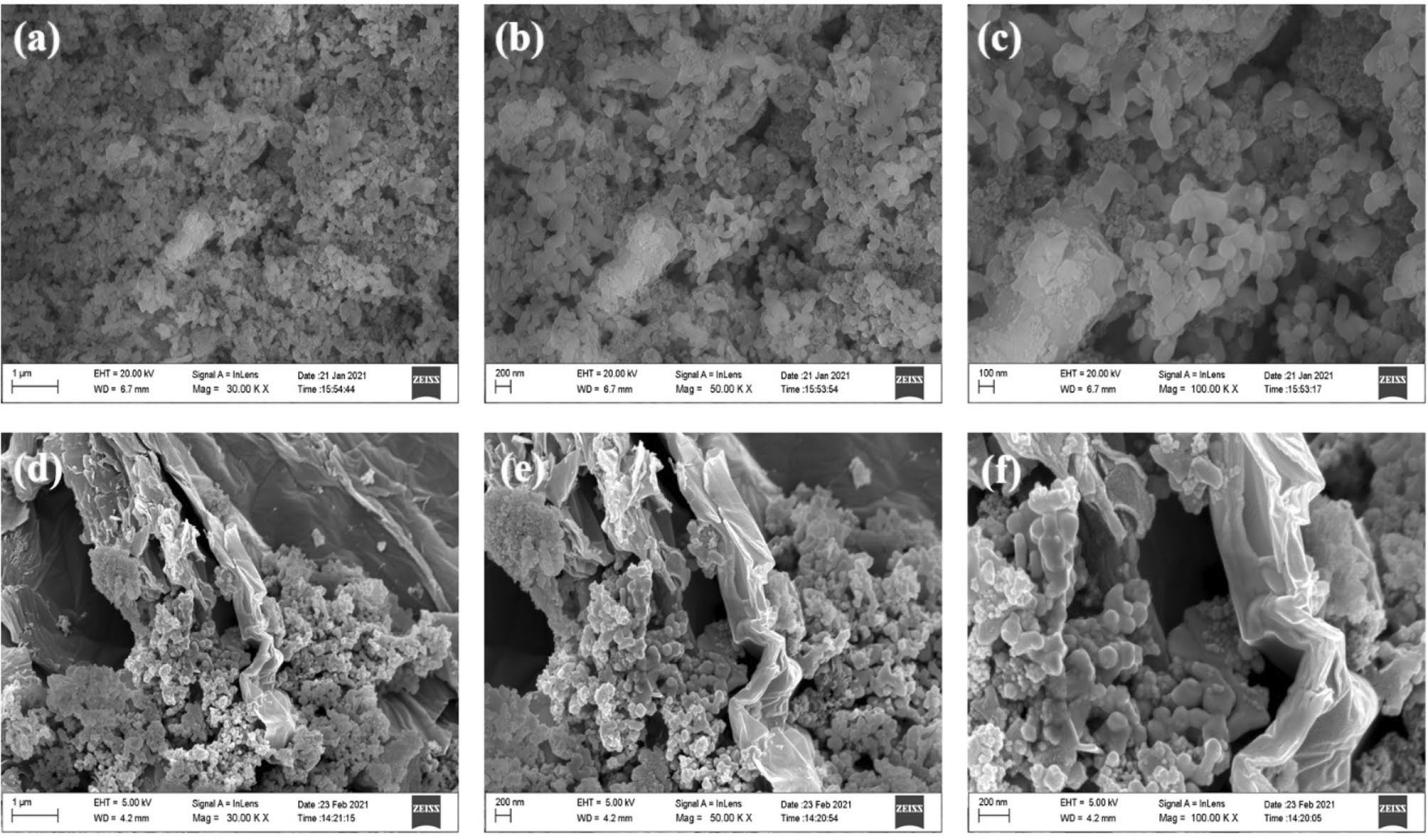
FE-SEM micrographs of Al_2_O_3_ at various magnifications (**a**) 30.00 KX, (**b**) 50.00 KX and (**c**) 100.00 KX. FE-SEM micrographs of Al_2_O_3_-reduced graphene oxide hybrid at various magnifications (**d**) 30.00 KX, (**e**) 50.00 KX and (**f**) 100.00 KX.

**Figure 4:**
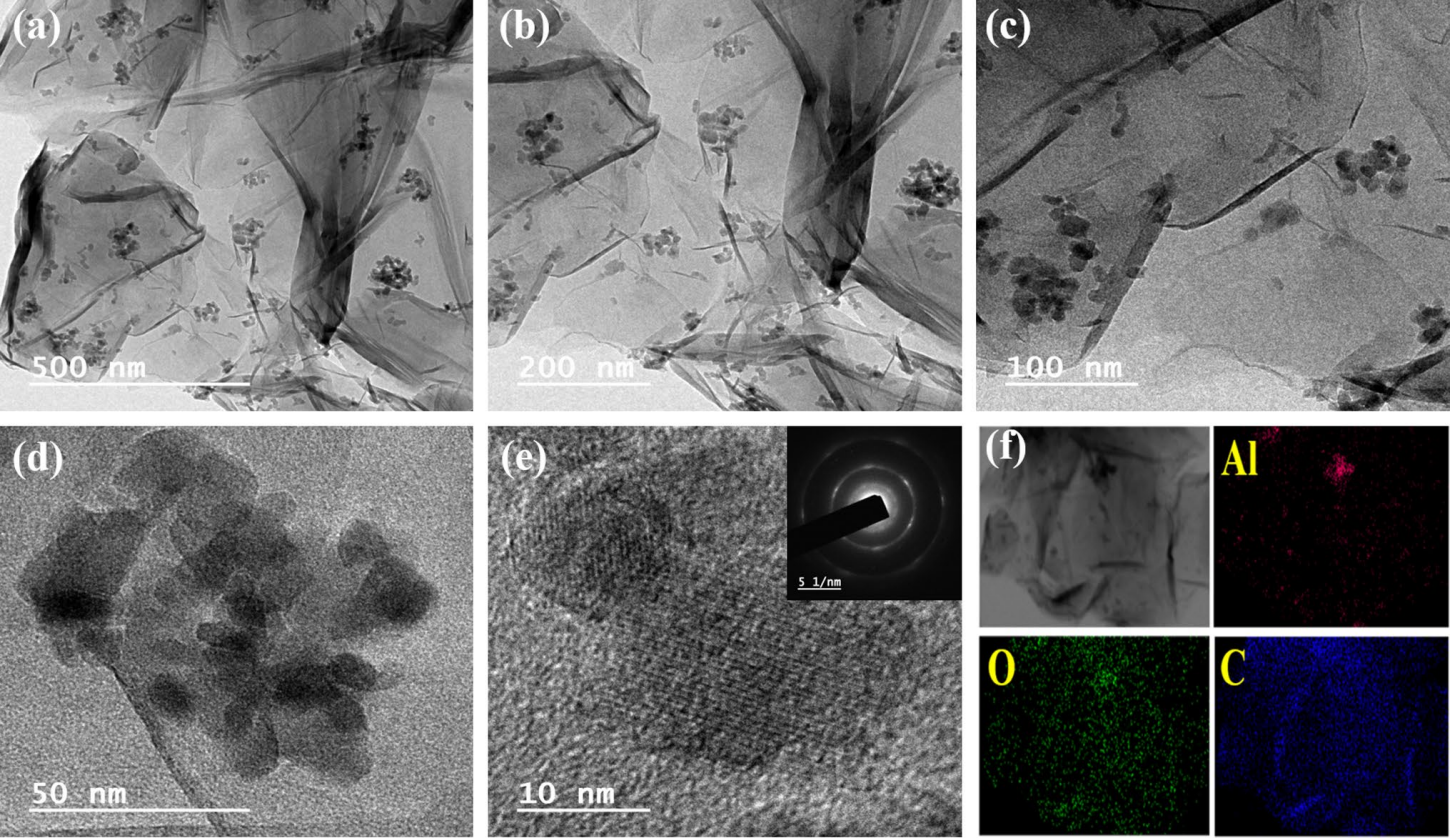
HR-TEM micrographs of Al_2_O_3_-reduced graphene oxide hybrid taken at various magnifications (500 nm (**a**), 200 nm (**b**), 100 nm (**c**), 50 nm (**d**) and 10 nm (**e**)). The inset in (**e**) contains the SAED pattern of Al_2_O_3_-reduced graphene oxide hybrid. (**f**) Elemental mapping of Al_2_O_3_-reduced graphene oxide hybrid during acquisition of the HR-TEM measurment (shows the presence of aluminium, oxygen and carbon element).

The micro-scale surface investigation of the samples was done with the help of FE-SEM, which is depicted in Fig. 3. Figure 3a–c shows the FE-SEM micrograph of Al_2_O_3,_ which reveals the formation of nanoparticles with a typical size in the range of 30–40 nm, evident through their respective high and low magnification micrographs. The FE-SEM micrographs for the Al_2_O_3_-reduced graphene oxide hybrid are provided in Fig. 3d–f and Fig. S2, indicating that Al_2_O_3_ nanoparticles are well decorated on the surface of the reduced graphene oxide nanosheet. The hybridisation of Al_2_O_3_, with reduced graphene oxide as a substrate, provides a highly conductive platform, enhancing the charge transfer kinetics during the electrochemical processes. The charge transfer occurs from highly conductive reduced graphene oxide to the Al_2_O_3_ nanoparticles, enhancing their pseudocapacitive performance, as evidenced by the obtained results^35^. The energy dispersive X-ray spectroscopy (EDX) and elemental mapping of Al_2_O_3_ and Al_2_O_3_-reduced graphene oxide hybrid have been illustrated in Figs. S3 and S4, respectively. The EDX spectroscopy indicates the presence of elements such as Al, O, and Al, O, C in Al_2_O_3_ and Al_2_O_3_-reduced graphene oxide hybrid nanostructure. The elemental mapping reveals the homogeneous distribution of elements such as Al, O (Fig. S3b–d) and Al, O, C (Fig. S4b–e) in the Al_2_O_3_ and Al_2_O_3_-reduced graphene oxide hybrid. Figure 4a–f represents the high-resolution transmission electron microscopic (HR-TEM) images (at various magnifications) and the elemental mapping obtained during the acquisition of the HR-TEM measurement of the Al_2_O_3_-reduced graphene oxide hybrid. The HR-TEM micrographs reveal that Al_2_O_3_ nanoparticles are decorated on reduced graphene oxide sheets, which agrees with the FE-SEM micrograph of the Al_2_O_3_-reduced graphene oxide hybrid. The inset of Fig. 4e shows the SAED pattern of the Al_2_O_3_-reduced graphene oxide hybrid. The existence of rings in the SAED pattern acknowledges that the Al_2_O_3_-reduced graphene oxide hybrid is polycrystalline^36^. The elemental mapping (Fig. 4f) indicates the uniform distribution of aluminium, oxygen, and carbon elements in the Al_2_O_3_-reduced graphene oxide hybrid.

**Figure 5:**
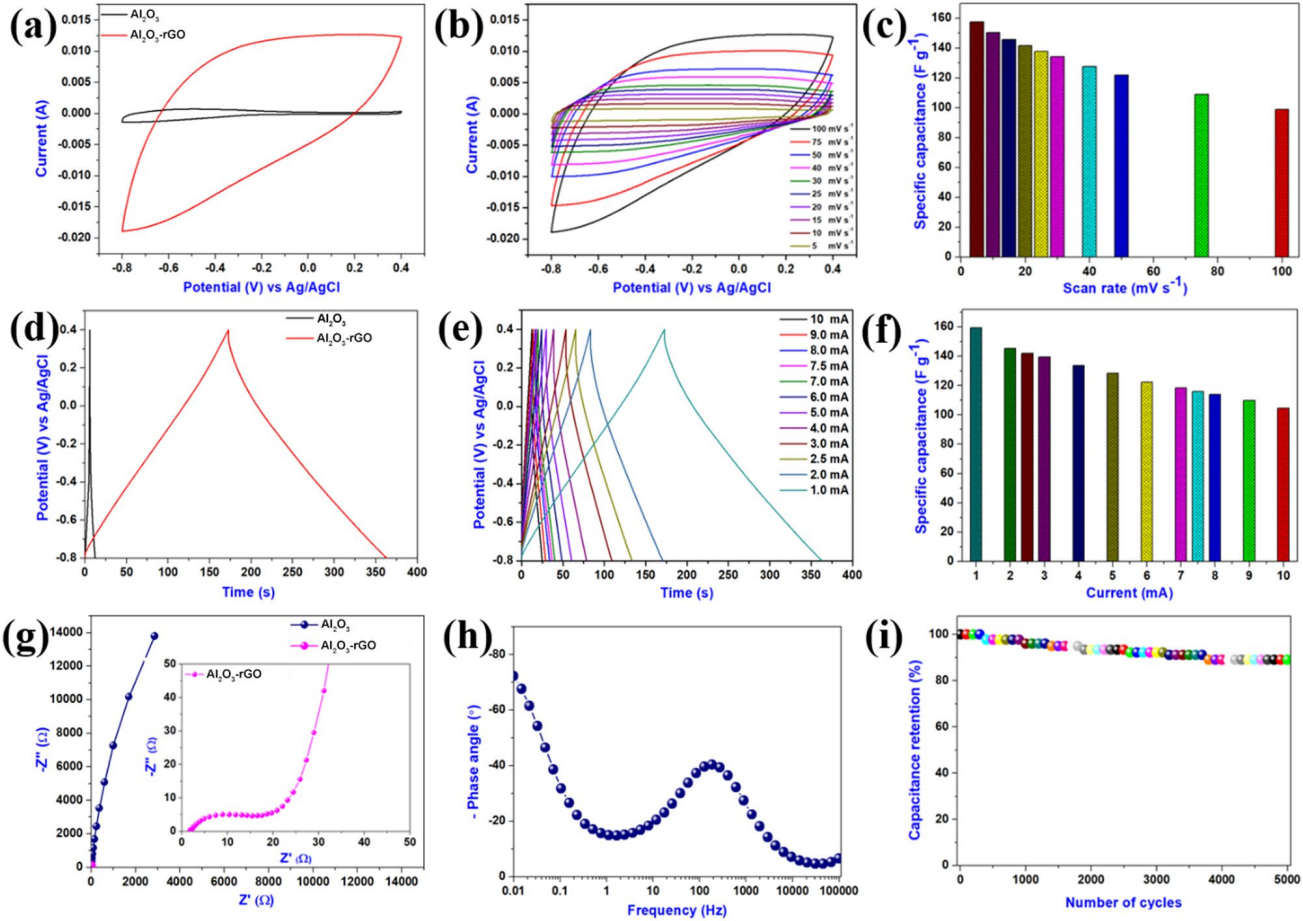
Electrochemical characterization of as synthesized Al_2_O_3_ and Al_2_O_3_-reduced graphene oxide hybrid. (**a**) Comparative cyclic voltammetric profiles of Al_2_O_3_ and Al_2_O_3_-reduced graphene oxide hybrid electrode recorded at a sweep rate of 100 mV s^−1^, (**b**) CV plots of Al_2_O_3_-reduced graphene oxide hybrid electrode recorded at different sweep rates, (**c**) effect of sweep rates on gravimetric capacitances of Al_2_O_3_-reduced graphene oxide hybrid electrode, (**d**) comparative charge–discharge profile of Al_2_O_3_ and Al_2_O_3_-reduced graphene oxide hybrid electrode recorded at applied current of 1 mA, (**e**) CD profile of Al_2_O_3_-reduced graphene oxide hybrid electrode recorded at various applied currents, (**f**) effect of applied currents on specific capacitances of Al_2_O_3_-reduced graphene oxide hybrid electrode, (**g**) comparative Nyquist plot Al_2_O_3_ and Al_2_O_3_-reduced graphene oxide hybrid electrode with inset shows the enlarged portion of the Nyquist plot of the Al_2_O_3_-reduced graphene oxide hybrid electrode, (**h**) Bode phase angle plot of Al_2_O_3_-reduced graphene oxide hybrid electrode, and (**i**) the cyclic stability performance of Al_2_O_3_-reduced graphene oxide hybrid electrode over 5000 cycles.

Electrochemical characteristics of bare Al_2_O_3_ and Al_2_O_3_-reduced graphene oxide hybrid electrodes were compared in a standard three-electrode electrochemical set-up, using one molar aqueous solution of Na_2_SO_4_ as the electrolyte. Figure 5a represents the comparative CV profile of Al_2_O_3_ and Al_2_O_3_-reduced graphene oxide hybrid at a scan rate of 100 mV s^−1^, within a potential range of − 0.8 to 0.4 V (vs. Ag/AgCl). It is intriguing to note that the Al_2_O_3_-reduced graphene oxide hybrid electrode can work at a much higher current range than the Al_2_O_3_ electrode, demonstrating the improved electrochemical performance of the Al_2_O_3_-reduced graphene oxide hybrid electrode. The improvement in the electrochemical performance of the Al_2_O_3_-reduced graphene oxide hybrid electrode compared to the bare Al_2_O_3_ electrode is due to the additional charge adsorption that occurs in the presence of an EDLC component like reduced graphene oxide content besides the pseudocapacitive contribution from the Al_2_O_3_. Also, the Al_2_O_3_-reduced graphene oxide hybrid electrode possesses a better electrode and electrolyte interface^37,38^. Figure 5b shows the CV profile of the Al_2_O_3_-reduced graphene oxide hybrid electrode at various sweep rates (5–100 mV s^−1^) in the potential range of − 0.8 to 0.4 V (vs. Ag/AgCl). The CV profile of the Al_2_O_3_-reduced graphene oxide hybrid electrode takes a quasi-rectangular shape, indicating the effective intralayer charge transfer and a nearly capacitive behaviour^39^. The effect of scan rate on the specific capacitance of the Al_2_O_3_-reduced graphene oxide hybrid electrode is shown in Fig. 5c. The Al_2_O_3_-reduced graphene oxide hybrid electrode exhibits the highest gravimetric capacitance, 157.29 F g^−1^, at 5 mV s^−1^, which is ~ 15-fold higher than the bare Al_2_O_3_ electrode (11.06 F g^−1^ at 5 mV s^−1^). The specific capacitance of the Al_2_O_3_-reduced graphene oxide hybrid electrode reduces at faster potential sweep rates, which can be attributed to the slower reaction kinetics and sluggish ion transportation inside the electrolytic medium^40,41^. The Al_2_O_3_-reduced graphene oxide hybrid retains about 62.76% of its initial capacitance, even when the potential sweep rate is increased 20-fold (100 mV s^−1^), indicating better rate performance of the Al_2_O_3_-reduced graphene oxide hybrid electrode.

Moreover, Al_2_O_3_ and Al_2_O_3_-reduced graphene oxide hybrid electrodes' electrochemical behaviour was also quantitatively determined from the CD curves. Figure 5d represents the comparative CD profiles of Al_2_O_3_ and Al_2_O_3_-reduced graphene oxide hybrid electrodes, which indicate that Al_2_O_3_-reduced graphene oxide hybrid electrodes need higher charge and discharge times than Al_2_O_3_ electrodes, thus suggesting improved electrochemical performance. The CD characteristics of the Al_2_O_3_-reduced graphene oxide hybrid electrode at various applied currents (1–10 mA) are recorded and shown in Fig. 5e. The CD curves exhibit nearly symmetric behaviour, signifying excellent reversibility. The charge storage phenomenon is a combination of EDLC and pseudocapacitive behaviour of the Al_2_O_3_-reduced graphene oxide hybrid electrode, which agrees with the CV profile^42^. Figure 5f represents the effect of different applied currents on the specific capacitance. The Al_2_O_3_-reduced graphene oxide hybrid electrode obtained a maximum specific capacitance of about 159.28 F g^−1^ at an applied current of 1 mA, which is quite high compared to the bare Al_2_O_3_ electrode (10.38 F g^−1^). The electrochemical performance of the Al_2_O_3_-reduced graphene oxide hybrid electrode is found to be higher than various electroactive materials listed in Table S1.

EIS measurements were performed using Nyquist and Bode plots in the frequency-dependent mode to verify the superior electrochemical behaviour of the Al_2_O_3_-reduced graphene oxide hybrid electrode over the bare Al_2_O_3_ electrode^43^. Figure 5g shows the Nyquist plots for Al_2_O_3_ and Al_2_O_3_-reduced graphene oxide hybrid electrodes, recorded in the frequency range of 0.01 Hz to 100 kHz. It is visible that the Al_2_O_3_-reduced graphene oxide hybrid electrode has a lower Warburg region (the straight line at low frequency) compared to the bare Al_2_O_3_ electrode, with solution resistance (*Rs*) and charge transfer resistance (*Rct*) values, 1.33 Ω and 16.67 Ω, respectively. The Al_2_O_3_-reduced graphene oxide hybrid electrode obtained a short diffusion path length for ion transfer, which could be noticed from the low resistance of the capacitive part on the Nyquist plot^44^. The Nyquist plot reveals that the Al_2_O_3_-reduced graphene oxide hybrid electrode has a higher electrical conductivity than the bare Al_2_O_3_ electrode due to the inclusion of reduced graphene oxide, which greatly enhanced the overall electron transfer rate^45^. The Bode phase angle plot of the Al_2_O_3_-reduced graphene oxide hybrid electrode is shown in Fig. 5h. The Bode phase angle of the Al_2_O_3_-reduced graphene oxide hybrid electrode tail at the low-frequency region is about − 72.29°, which suggests that the charge storage occurs in terms of both EDLC and pseudocapacitive activities in the hybrid^46^. The electrode was then subject to continuous CD measurement for over 5000 cycles at a constant charge–discharge current of 7.5 mA, which is shown in Fig. 5i. The Al_2_O_3_-reduced graphene oxide hybrid electrode retained ~ 89% of the initial specific capacitance after 5000 CD cycles. To investigate the specific capacitance decay in the Al_2_O_3_-reduced graphene oxide hybrid electrode, we executed EIS measurement using the Nyquist plot before and after cyclic stability and presented in Fig. S5. There is a slight variation in the value of *Rs* (1.33 to 1.43) and *Rct* (16.67 to 23.07) values before and after the cyclic stability test, which may be the reason behind the observed capacitance decay. Detailed CV and CD tests were carried out on the bare Al_2_O_3_ electrode at various scan rates and normalised current values, as shown in Fig. S6. The variation of specific capacity to the applied current of bare Al_2_O_3_ electrode and Al_2_O_3_-reduced graphene oxide hybrid electrode is provided in Fig. S7^47,48^. To further identify the charge storage or capacitance contribution by EDLC and pseudocapacitance process, the Trasatti plot of the bare Al_2_O_3_ electrode and Al_2_O_3_-reduced graphene oxide hybrid electrode has been provided in Fig. S8^49,50^. The overall capacitance of the electroactive material can be ascribed to the combined effect of the surface adsorbed electric double-layer process and the diffusion-controlled pseudocapacitive process. In Fig. S8a,b, the y-intercept of the linear fit of 1/C_sp_ vs. v^1/2^ at v = 0 indicates the total charge stored in the bare Al_2_O_3_ electrode and Al_2_O_3_-reduced graphene oxide hybrid electrode, respectively. Similarly, in Fig. S8c,d, the y-intercept of the linear fit of C_csp_ vs. v^-1/2^ at v = ∞ illustrates the amount of charge stored due to the electric double-layer process in the bare Al_2_O_3_ electrode and Al_2_O_3_-reduced graphene oxide hybrid electrode, respectively. The pseudocapacitive contribution can be estimated by subtracting the electric double-layer capacitance from the overall capacitance. According to the Trasatti method (as shown in Fig. S8e), the pseudocapacitive contributions of the bare Al_2_O_3_ electrode and Al_2_O_3_-reduced graphene oxide hybrid electrode were found to be 80.25% and 50.74%.

In contrast, the electric double-layer contributions are 19.75% and 49.26%, respectively. The higher electrochemical performance of the Al_2_O_3_-reduced graphene oxide hybrid electrode, compared to the bare Al_2_O_3_ electrode, can be attributed to the constructive synergistic effects caused by the attachment of Al_2_O_3_ nanoparticles to the reduced graphene oxide nanosheets. In the Al_2_O_3_-reduced graphene oxide hybrid electrode, the open space inside the reduced graphene oxide nanosheets for Al_2_O_3_ nanoparticles offers an easy path for electrolyte diffusion during the electrochemical analysis, which leads to higher electronic and ionic conduction, resulting in higher electrochemical performance compared to bare Al_2_O_3_ electrode^51,52^.

**Figure 6:**
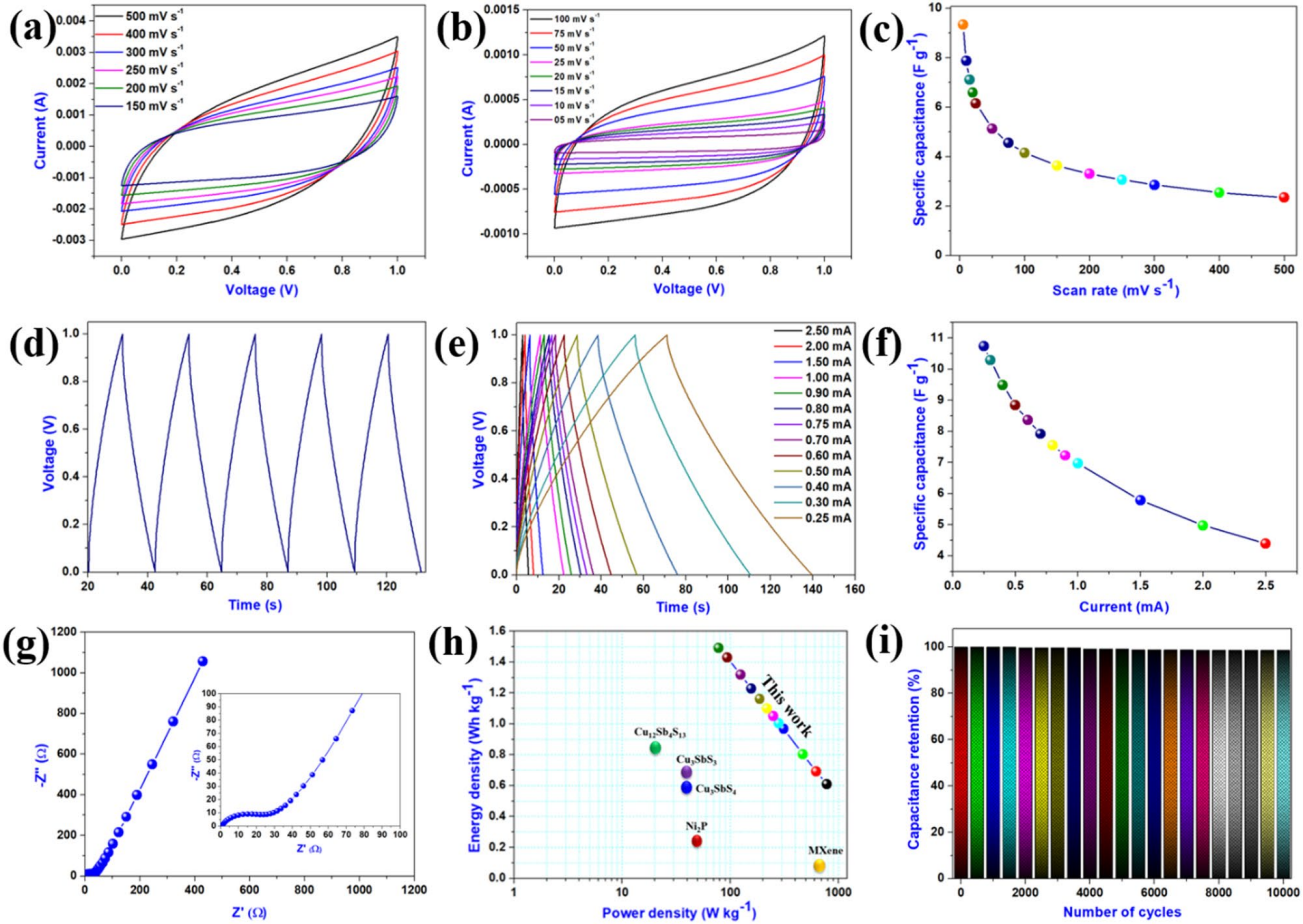
Electrochemical characterization of Al_2_O_3_-reduced graphene oxide hybrid symmetric cell SSD. Cyclic voltammetric profiles Al_2_O_3_-reduced graphene oxide hybrid symmetric cell SSD at different sweep rates (**a**) 150–500 mV s^−1^, (**b**) 5–100 mV s^−1^, (**c**) effect of sweep rates on gravimetric capacitances of Al_2_O_3_-reduced graphene oxide hybrid symmetric cell SSD of Al_2_O_3_-reduced graphene oxide hybrid symmetric cell SSD, (**d**) charge–discharge profile of Al_2_O_3_-reduced graphene oxide hybrid symmetric cell SSD recorded at applied current of 1 mA, (**e**) CD plots of Al_2_O_3_-reduced graphene oxide hybrid symmetric cell SSD recorded at various currents, (**f**) effect of applied currents on specific capacitances of Al_2_O_3_-reduced graphene oxide hybrid symmetric cell SSD, (**g**) Nyquist plot of Al_2_O_3_-reduced graphene oxide hybrid symmetric cell SSD with inset shows the enlarged view, (**h**) Ragone plot of Al_2_O_3_-reduced graphene oxide hybrid symmetric cell SSD with other reported SSD, and (**i**) cyclic stability performance of Al_2_O_3_-reduced graphene oxide hybrid symmetric cell SSD over 10,000 cycles.

A supercapacitor device was fabricated (in symmetric configuration), using Al_2_O_3_-reduced graphene oxide hybrid electrode as both the negative and positive electrodes to examine the potential of the Al_2_O_3_-reduced graphene oxide hybrid electrode for real-world applications. The device was tested in 1 M aqueous Na_2_SO_4_ electrolyte within the potential range of 1 V. The detailed electrochemical performances of the assembled symmetric device have been illustrated in Fig. 6. Figure 6a,b illustrates the CV profiles of Al_2_O_3_-reduced graphene oxide hybrid SSD, recorded at various sweep rates (5–500 mV s^−1^) in the potential range of 0.0–1.0 V. The CV profiles of Al_2_O_3_-reduced graphene oxide hybrid SSD demonstrate typical rectangular behaviour, which remains almost the same with the increase in the sweep rate from 5 to 500 mV s^−1^, indicating the capacitive characteristic^53^. The specific capacitance values of Al_2_O_3_-reduced graphene oxide hybrid SSD at various scan rates are shown in Fig. 6c. The highest specific capacitance of Al_2_O_3_-reduced graphene oxide hybrid SSD has been calculated to be about 9.34 F g^−1^ at the scan rate of 5 mV s^−1^. The CD curves of the Al_2_O_3_-reduced graphene oxide hybrid SSD are shown in Fig. 6d,e. Figure 6d and e represent the CD profiles of Al_2_O_3_-reduced graphene oxide hybrid SSD at a constant current of 1 mA and various applied currents from 0.25 to 2.50 mA. The CD profiles reveal that the charging and discharging profiles of CD curves possess good symmetry. The graph between gravimetric capacitance and different applied current values for Al_2_O_3_-reduced graphene oxide hybrid SSD is shown in Fig. 6f. It was observed that the highest specific capacitance of about 10.73 F g^−1^ can be obtained at an applied current of 0.25 mA. To further understand the charge transfer kinetics in the case of Al_2_O_3_-reduced graphene oxide hybrid SSD, EIS measurement was performed in the frequency range of 0.01–100 kHz, using the Nyquist and Bode plot. Figure 6g represents the Nyquist plot for Al_2_O_3_-reduced graphene oxide hybrid SSD, with an inset showing the Nyquist plot's enlarged view at the low-frequency region. The Nyquist analysis shows that the values of *Rs* and *Rct* are about 0.81 Ω and 23.55 Ω, respectively, indicating good electrical conductivity of the Al_2_O_3_-reduced graphene oxide hybrid SSD. The Bode plot (phase angle as a function of frequency) of Al_2_O_3_-reduced graphene oxide hybrid SSD is shown in Fig. S9. The Bode plot reveals that the phase angle of Al_2_O_3_-reduced graphene oxide hybrid SSD is about -67.90°, which signifies both EDLC and pseudocapacitive contributions^46^. To describe the specific energy and specific power characteristics of the Al_2_O_3_-reduced graphene oxide hybrid SSD, Fig. 6h represents the Ragone plot for the as-fabricated SSD. The Al_2_O_3_-reduced graphene oxide hybrid SSD obtained a maximum energy density of about 1.49 Wh kg^−1^ with a corresponding power density of 78.125 W kg^−1^. The power density of SSD increases up to 1.562 kW kg^−1^ with an increased applied current of 5 mA. The Ragone plot clearly indicates higher specific energy of Al_2_O_3_-reduced graphene oxide hybrid SSD compared to Ni_2_P (0.24 Wh kg^−1^)^54^, Cu_3_SbS_4_ (0.64 Wh kg^−1^), Cu_3_SbS_3_ (0.7 Wh kg^−1^), Cu_12_Sb_4_S_13_ (0.85 Wh kg^−1^)^55^, Mxene (0.089 Wh kg^−1^)^56^. Figure 6i represents the cyclic stability plot for Al_2_O_3_-reduced graphene oxide hybrid SSD, obtained at a constant current of 1 mA for over 10,000 cycles. The Al_2_O_3_-reduced graphene oxide hybrid SSD demonstrates excellent cyclic stability with a retention of 98.56%. The obtained capacitance retention (98.56%) of Al_2_O_3_-reduced graphene oxide hybrid SSD is higher compared to the previously reported SSD such as rGO-PMo_12_ (89–95%)^57^, Co_3_O_4_-VAGN (86.3%)^58^, MXene-reduced graphene oxide (90%)^59^, cobalt hexacyanoferrate-reduced graphene oxide (83%)^60^ and famatinite-reduced graphene oxide (95.5%)^61^. To support the excellent cyclic stability of Al_2_O_3_-reduced graphene oxide hybrid SSD, we performed EIS analyses before and after 10,000 cycles and found no such variation in *Rs* and *Rct* values after the cyclic stability test, as shown in Fig. S10. To further clarify the excellent cyclic stability, we have provided the first and last ten cycles of 10,000 cycles (as shown in Fig. S11), which reveals no such changes in the time duration of charging and discharging profiles of Al_2_O_3_-reduced graphene oxide hybrid SSD. To demonstrate the real-time application of the Al_2_O_3_-reduced graphene oxide hybrid, we have fabricated Al_2_O_3_-reduced graphene oxide hybrid based coin cell SSD and successfully powered a red LED by series connection (as shown in Fig. S12).

### Structure and electronic properties

**Figure 7:**
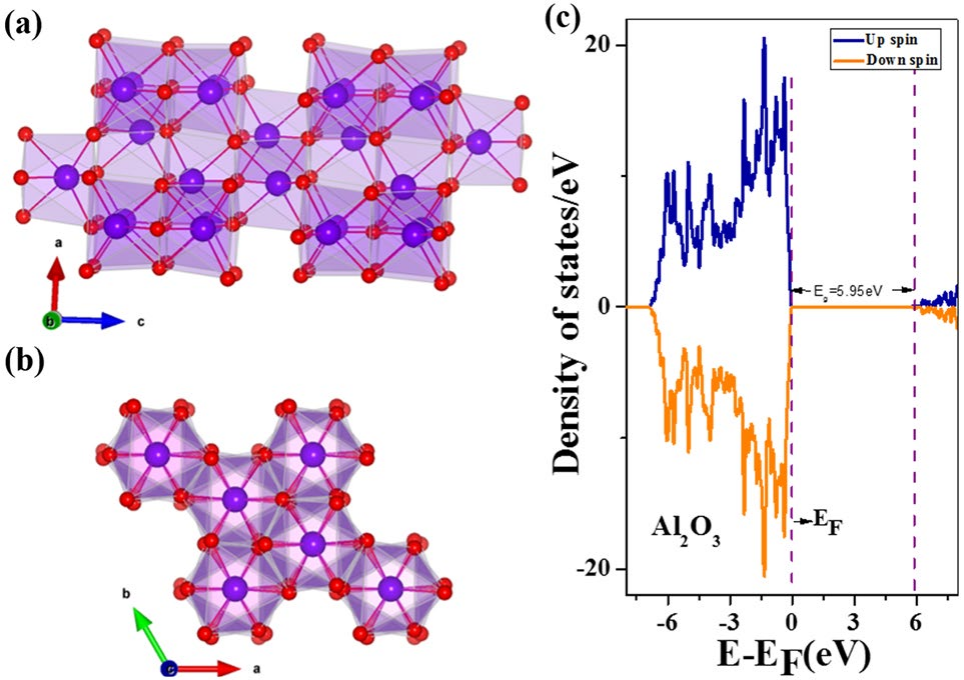
(**a**, **b**) DFT optimized structure for bulk Al_2_O_3_. (**c**) Total Density of States for bulk Al_2_O_3_. Fermi level is at 0 eV and indicated by dotted line.

**Figure 8:**
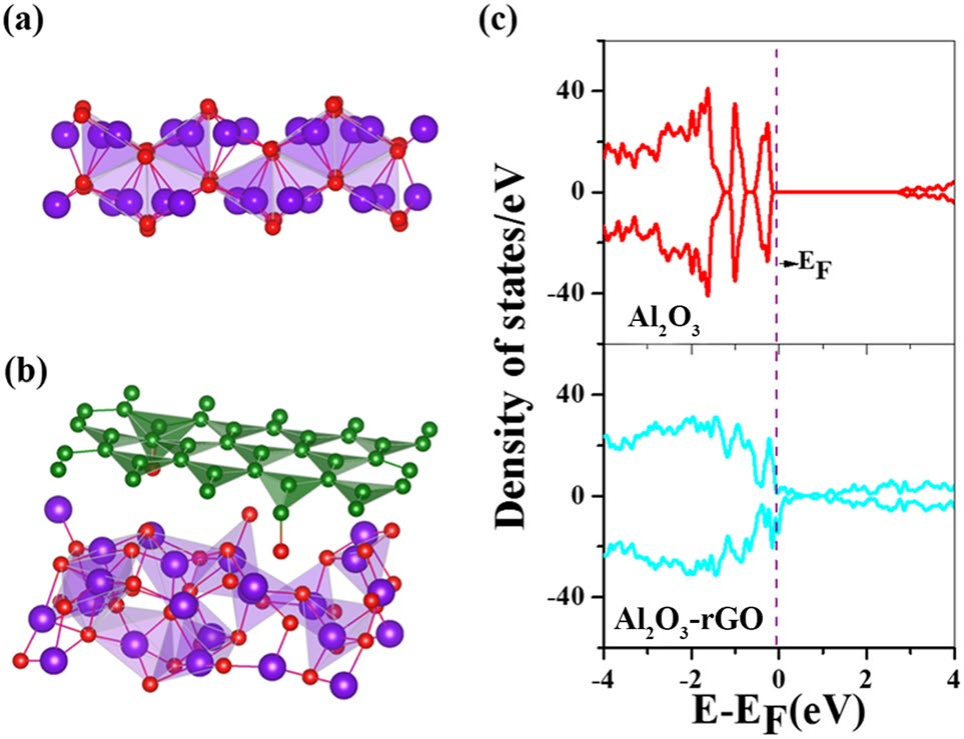
(**a**) DFT optimized structure for generated (113) surface of Al_2_O_3_, (**b**) DFT optimized structure for hybrid layer Al_2_O_3_-reduced graphene oxide, (**c**) Total Density of States for (113) surface of Al_2_O_3_(upper panel) and for hybrid layer Al_2_O_3_-reduced graphene oxide (lower panel). Fermi level is at 0 eV and indicated by dotted line.

We have considered Al_2_O_3_ having space group R-3C with the hexagonal unit cell containing 12 six-coordinated Al atoms and 18 four-coordinated O atoms. The relaxed bulk structure is displayed in Fig. 7a,b. DFT optimized lattice parameters are a = b = 4.80304 Ẳ, c = 13.11089 Ẳ, α = β = 90, γ = 120 degree. Computed lattice parameters are in good agreement with the experimental data from the literature, a = b = 4.77 Ẳ, c = 13.01 Ẳ^62^. Figure 7c presents the density of states for bulk Al_2_O_3_. The symmetry between up-spin and down-spin channels indicates a non-magnetic signature where a large gap at the Fermi level indicates that Al_2_O_3_ is a wide band gap insulator. The computed band gap employing GGA exchange–correlation functional is 5.95 eV, matching well with PBE-based theoretical data of 6.045 eV from literature^63^. But, it is lower than the experimental value of 8.8 eV as DFT underestimates the band gap and more sophisticated methods like hybrid functional calculations are needed to match experimental data^64^. Using the relaxed structure of bulk Al_2_O_3,_ we have generated (113) surface of Al_2_O_3_ since it has the most intense XRD peak. Further, we allowed the (113) surface of Al_2_O_3_ to relax for minimum energy configuration, and Fig. 8a depicts the relaxed surface of Al_2_O_3._ From the relaxed (113) surface of the Al_2_O_3_ and reduced graphene oxide layer, we have generated a hybrid layer of Al_2_O_3_-reduced graphene oxide so that the lattice mismatch is negligible. The geometry-optimized hybrid layer of Al_2_O_3_-reduced graphene oxide is displayed in Fig. 8b. We have plotted the total electronic density of states for (113) plane Al_2_O_3_ and the hybrid layer of Al_2_O_3_-reduced graphene oxide in Fig. 8c. We can observe that the hybrid layer becomes metallic, and there is an enhancement in electronic states near the Fermi level, which indicates enhanced conductivity of the system.

### Orbital interaction and charge transfer

**Figure 9:**
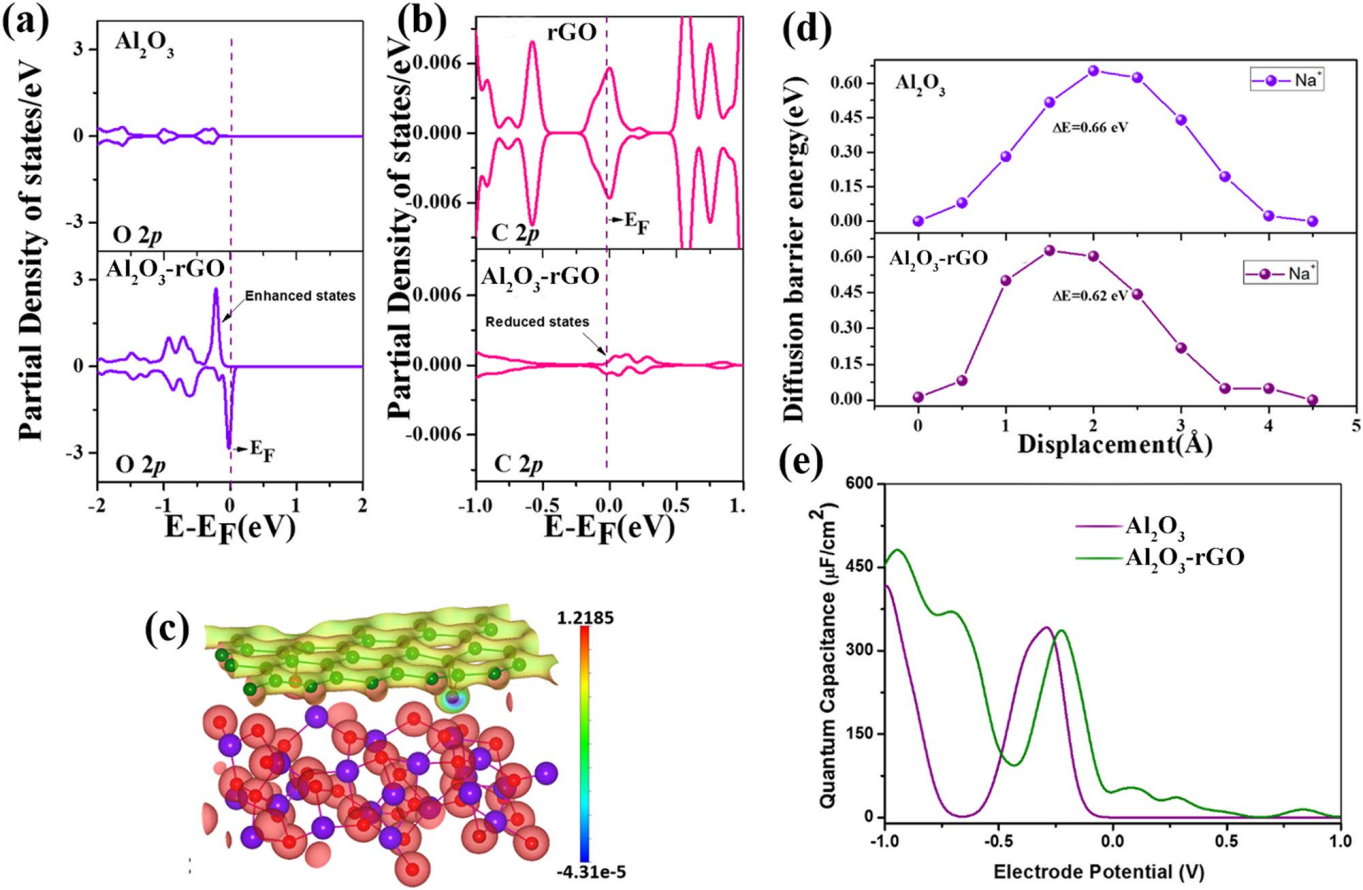
(**a**) Partial Density of States for O 2p orbital of Al_2_O_3_ for bare Al_2_O_3_ and hybrid Al_2_O_3_-reduced graphene oxide, (**b**) Partial Density of States for C 2p orbital of reduced graphene oxide for pristine reduced graphene oxide and hybrid Al_2_O_3_-reduced graphene oxide; Fermi level is at 0 eV and indicated by dotted line, (**c**) charge density plot for the charge density difference between Al_2_O_3_-reduced graphene oxide and Al_2_O_3_, (**d**) diffusion energy barrier of electrolytic ions in bare Al_2_O_3_ and hybrid Al_2_O_3_-reduced graphene oxide, (**e**) The change in the quantum capacitance with the variation in the electrode potential for in bare Al_2_O_3_ and Al_2_O_3_-reduced graphene oxide hybrid.

To find the orbital interactions between Al_2_O_3_ and reduced graphene oxide, in Fig. 9a–c, we have presented the partial density of states for the O 2p orbital of Al_2_O_3_ and C 2p orbital of reduced graphene oxide for the hybrid system and corresponding pristine cases. We can notice that for the O 2p orbital of Al_2_O_3,_ there is an enhancement in the electronic states in the hybrid system compared to pure Al_2_O_3_. Similarly, for the C 2p orbital of reduced graphene oxide, there is a reduction in the electronic states near the Fermi level in the hybrid system compared to pristine reduced graphene oxide. This enhancement in states for O 2p orbital and reduction in states for C 2p orbital of reduced graphene oxide indicate that the interaction is due to charge transfer from reduced graphene oxide to Al_2_O_3_. To find the quantitative charge transfer, we have performed Bader charge analysis. According to Bader charge partitioning, Al_2_O_3_ has a gain of 0.38e charge in the supercell. The difference in the charge density between Al_2_O_3_-reduced graphene oxide and Al_2_O_3_ has been plotted in Fig. 9c to visualize the spatial variation of electronic charge. The charge-gaining region corresponds to Al_2_O_3_ and is denoted by red colour.

### Diffusion energy barrier of electrolytic ions

We have also computed the diffusion energy barrier of the electrolytic ion Na^+^ in Al_2_O_3_ and in hybrid Al_2_O_3_-reduced graphene oxide. The lower the diffusion energy barrier, the higher the mobility and the better the charge transfer capability. We can see from Fig. 9d that the diffusion energy barrier for Na^+^ ions is lower for the hybrid Al_2_O_3_-reduced graphene oxide system, justifying the superior charge storage performance in the hybrid system compared to Al_2_O_3_.

### Computation of quantum capacitance

Employing the electronic Density of States, we have calculated the quantum capacitance using the following relationship^65^.

5 $$C_{Q} = e^{2} \mathop \smallint \limits_{\infty }^{\infty } D\left( E \right)F_{T} \left( {E - e\phi_{G} } \right)dE$$

In the above equation, *D(E)* denotes the density of states, $\phi_{G}$ indicates electrode potential and the function, *F*_*T*_ (*E*), is the thermal broadening which can be written as;

6 $$F_{T} \left( E \right) = (4K_{B} T)^{ - 1} \sec h^{2} \left( {E/2K_{B} T} \right)$$

Figure 9e describes the variation between electrode potential and quantum capacitance. We can observe that for most of the electrode potential, the quantum capacitance is higher for the hybrid system Al_2_O_3_-reduced graphene oxide. Quantum capacitance is dominant for low dimensional systems, and the total capacitance is related to quantum capacitance through the relation^66^:

7 $$\frac{1}{{C_{T} }} = \frac{1}{{C_{Q} }} + \frac{1}{{C_{EDL} }}$$

*C*_*EDL*_ indicates electric double-layer capacitance, which depends on the electrode–electrolyte interfacial interaction. The increased quantum capacitance value in the hybrid material suggests higher charge storage performance for the Al_2_O_3_-reduced graphene oxide system than bare Al_2_O_3_.

## Conclusions

In summary, nanostructured Al_2_O_3_ and Al_2_O_3_-reduced graphene oxide hybrid were successfully synthesized through a two-step method comprising hydrothermal and post-hydrothermal calcination processes. Al_2_O_3_ and Al_2_O_3_-reduced graphene oxide hybrid electrodes' supercapacitor performances were evaluated through both theoretical and experimental methods for the first time. Due to the synergistic effects of both EDLC and pseudocapacitance, the Al_2_O_3_-reduced graphene oxide hybrid obtained higher electrochemical performance than the bare Al_2_O_3_ electrode. Remarkably, the Al_2_O_3_-reduced graphene oxide hybrid electrodes-based SSD manifests state-of-the-art capacitance retention of 98.56%, even after 10,000 cycles. Furthermore, we have presented the electronic properties, diffusion energy barrier, and quantum capacitance for Al_2_O_3_ and Al_2_O_3_-reduced graphene oxide hybrid using Density Functional Theory simulations. The interaction between Al_2_O_3_ and reduced graphene oxide is due to the charge transfer from reduced graphene oxide to Al_2_O_3_. The hybrid structure exhibits higher quantum capacitance and lower diffusion energy barrier for the electrolytic ions, which contribute to improved charge storage performance for the hybrid Al_2_O_3_-reduced graphene oxide system supporting our experimental observation. Therefore, this research might give some insights into exploring low-cost advanced electroactive materials for promising applications in energy storage devices with state-of-the-art performance metrics.

## Supplementary Information

Supplementary Information.

## Data Availability

The datasets generated and/or analysed during the current study are available from the corresponding author on reasonable request.

## References

[1] Kumar S (2021). 0D to 3D carbon-based networks combined with pseudocapacitive electrode material for high energy density supercapacitor: A review. Chem. Eng. J..

[2] Patra A (2021). Understanding the charge storage mechanism of supercapacitors: In situ/operando spectroscopic approaches and theoretical investigations. J. Mater. Chem. A.

[3] Kandasamy M, Sahoo S, Nayak SK, Chakraborty B, Rout CS (2021). Recent advances in engineered metal oxide nanostructures for supercapacitor applications: Experimental and theoretical aspects. J. Mater. Chem. A.

[4] Zhao J, Burke AF (2021). Review on supercapacitors: Technologies and performance evaluation. J. Energy Chem..

[5] Sahoo S, Ratha S, Rout CS, Nayak SK (2022). Self-charging supercapacitors for smart electronic devices: A concise review on the recent trends and future sustainability. J. Mater. Sci..

[6] Xu Y, Lu W, Xu G, Chou T-W (2021). Structural supercapacitor composites: A review. Compos. Sci. Technol..

[7] Hussain I (2021). Binder-free trimetallic phosphate nanosheets as an electrode: Theoretical and experimental investigation. J. Power Sources.

[8] Hussain I (2021). An oriented Ni–Co-MOF anchored on solution-free 1D CuO: Ap–n heterojunction for supercapacitive energy storage. J. Mater. Chem. A.

[9] Ramesh S, Khandelwal S, Rhee KY, Hui D (2018). Synergistic effect of reduced graphene oxide, CNT and metal oxides on cellulose matrix for supercapacitor applications. Compos. Part B Eng..

[10] Krishnan SG, Arulraj A, Khalid M, Reddy MV, Zhang R (2021). Energy storage in metal cobaltite electrodes: Opportunities & challenges in magnesium cobalt oxide. Renew. Sustain. Energy Rev..

[11] Yar A, Krishnan SG, Dennis JO, Khalid M, Jose R (2021). Template-assisted electrodeposited cupric oxide nanotubes and hierarchical nanospikes for tailoring electrode-electrolyte interfacial charge transfer. Ceram. Int..

[12] Huang Y (2018). Hierarchically mesostructured aluminum current collector for enhancing the performance of supercapacitors. ACS Appl. Mater. Interfaces.

[13] Hong S, Bae J, Koo B, Kim Y-B (2014). High-performance ultra-thin film solid oxide fuel cell using anodized-aluminum-oxide supporting structure. Electrochem. commun..

[14] Kedzierski MA, Brignoli R, Quine KT, Brown JS (2017). Viscosity, density, and thermal conductivity of aluminum oxide and zinc oxide nanolubricants. Int. J. Refrig..

[15] Wei N, Hu J, Zhang M, He J, Ni P (2019). Cross-linked porous polymer separator using vinyl-modified aluminum oxide nanoparticles as cross-linker for lithium-ion batteries. Electrochim. Acta.

[16] Di S, Gong L, Zhou B (2020). Precipitated synthesis of Al2O3-ZnO nanorod for high-performance symmetrical supercapacitors. Mater. Chem. Phys..

[17] Maharana B (2021). High charge-storage performance of morphologically modified anatase TiO_2_: Experimental and theoretical insight. Phys. Rev. Appl..

[18] Sahoo S, Sahoo G, Jeong SM, Rout CS (2022). A review on supercapacitors based on plasma enhanced chemical vapor deposited vertical graphene arrays. J. Energy Storage.

[19] Zhao B (2017). A high-energy, long cycle-life hybrid supercapacitor based on graphene composite electrodes. Energy Storage Mater..

[20] Xiong C (2019). The recent progress on three-dimensional porous graphene-based hybrid structure for supercapacitor. Compos. Part B Eng..

[21] Sahoo S (2020). Hydrothermally synthesized chalcopyrite platelets as electrode material for symmetric supercapacitors. Inorg. Chem. Front..

[22] Kresse G, Furthmüller J (1996). Efficient iterative schemes for ab initio total-energy calculations using a plane-wave basis set. Phys. Rev. B.

[23] Kresse G, Joubert D (1999). From ultrasoft pseudopotentials to the projector augmented-wave method. Phys. Rev. b.

[24] Blöchl PE (1994). Projector augmented-wave method. Phys. Rev. B.

[25] Perdew JP, Burke K, Ernzerhof M (1996). Generalized gradient approximation made simple. Phys. Rev. Lett..

[26] Perdew JP (1992). Atoms, molecules, solids, and surfaces: Applications of the generalized gradient approximation for exchange and correlation. Phys. Rev. B.

[27] Monkhorst HJ, Pack JD (1976). Special points for Brillouin-zone integrations. Phys. Rev. B.

[28] Li, Z., Wu, K., Cao, J. & Wang, Y. Controlled synthesis of α-Al2O3 via the hydrothermal-pyrolysis method. in *IOP Conference Series: Materials Science and Engineering* vol. 207 12004 (IOP Publishing, 2017).

[29] Madito MJ (2021). Correlation of the graphene fermi-level shift and the enhanced electrochemical performance of graphene-manganese phosphate for hybrid supercapacitors: Raman spectroscopy analysis. ACS Appl. Mater. Interfaces.

[30] Kwan YCG, Ng GM, Huan CHA (2015). Identification of functional groups and determination of carboxyl formation temperature in graphene oxide using the XPS O 1s spectrum. Thin Solid Films.

[31] Jiang Z, Wang J, Meng L, Huang Y, Liu L (2011). A highly efficient chemical sensor material for ethanol: Al_2_O_3_/Graphene nanocomposites fabricated from graphene oxide. Chem. Commun..

[32] Jankovský O (2014). Towards highly electrically conductive and thermally insulating graphene nanocomposites: Al_2_O_3_–graphene. RSC Adv..

[33] Deng L, Liu J, Ma Z, Fan G, Liu Z (2018). Free-standing graphene/bismuth vanadate monolith composite as a binder-free electrode for symmetrical supercapacitors. RSC Adv..

[34] Rameshbabu R, Vinoth R, Navaneethan M, Hayakawa Y, Neppolian B (2017). Fabrication of Cu_2_MoS_4_ hollow nanotubes with rGO sheets for enhanced visible light photocatalytic performance. CrystEngComm.

[35] Sahoo S, Krishnamoorthy K, Pazhamalai P, Mariappan VK, Kim S-J (2019). Copper molybdenum sulfide nanoparticles embedded on graphene sheets as advanced electrodes for wide temperature-tolerant supercapacitors. Inorg. Chem. Front..

[36] Mariappan VK (2019). Two dimensional famatinite sheets decorated on reduced graphene oxide: A novel electrode for high performance supercapacitors. J. Power Sources.

[37] He W (2017). Ultrathin and porous Ni_3_S_2_/CoNi_2_S4 3D-network structure for superhigh energy density asymmetric supercapacitors. Adv. Energy Mater..

[38] Stoller MD, Ruoff RS (2010). Best practice methods for determining an electrode material's performance for ultracapacitors. Energy Environ. Sci..

[39] Peng L (2013). Ultrathin two-dimensional MnO_2_/graphene hybrid nanostructures for high-performance, flexible planar supercapacitors. Nano Lett..

[40] Vinoth S, Subramani K, Ong W-J, Sathish M, Pandikumar A (2021). CoS2 engulfed ultra-thin S-doped g-C_3_N_4_ and its enhanced electrochemical performance in hybrid asymmetric supercapacitor. J. Colloid Interface Sci..

[41] Dubal DP, Abdel-Azeim S, Chodankar NR, Han Y-K (2019). Molybdenum nitride nanocrystals anchored on phosphorus-incorporated carbon fabric as a negative electrode for high-performance asymmetric pseudocapacitor. iScience.

[42] Liu Y, Jiang SP, Shao Z (2020). Intercalation pseudocapacitance in electrochemical energy storage: Recent advances in fundamental understanding and materials development. Mater. Today Adv..

[43] Navalpotro P, Anderson M, Marcilla R, Palma J (2018). Insights into the energy storage mechanism of hybrid supercapacitors with redox electrolytes by electrochemical impedance spectroscopy. Electrochim. Acta.

[44] Baig MM, Gul IH, Baig SM, Shahzad F (2021). The complementary advanced characterization and electrochemical techniques for electrode materials for supercapacitors. J. Energy Storage.

[45] Singh KP, Bhattacharjya D, Razmjooei F, Yu J-S (2016). Effect of pristine graphene incorporation on charge storage mechanism of three-dimensional graphene oxide: superior energy and power density retention. Sci. Rep..

[46] Qi JL (2015). Vertically oriented few-layer graphene-nanocup hybrid structured electrodes for high-performance supercapacitors. J. Mater. Chem. A.

[47] Hussain I, Hussain T, Lamiel C, Zhang K (2020). Turning indium oxide into high-performing electrode materials via cation substitution strategy: Preserving single crystalline cubic structure of 2D nanoflakes towards energy storage devices. J. Power Sources.

[48] Hussain I (2021). Integration of CuO nanosheets to Zn-Ni-Co oxide nanowire arrays for energy storage applications. Chem. Eng. J..

[49] Sathiya M, Prakash AS, Ramesha K, Tarascon JM, Shukla AK (2011). V2O5-anchored carbon nanotubes for enhanced electrochemical energy storage. J. Am. Chem. Soc..

[50] Sankar KV, Selvan RK, Meyrick D (2015). Electrochemical performances of CoFe_2_O_4_ nanoparticles and a rGO based asymmetric supercapacitor. RSC Adv..

[51] Ma W (2016). Hierarchical MnO_2_ nanowire/graphene hybrid fibers with excellent electrochemical performance for flexible solid-state supercapacitors. J. Power Sources.

[52] Naderi HR, Norouzi P, Ganjali MR (2016). Electrochemical study of a novel high performance supercapacitor based on MnO2/nitrogen-doped graphene nanocomposite. Appl. Surf. Sci..

[53] Zhang, S. & Pan, N. Supercapacitors performance evaluation. *Adv. Energy Mater.* **5**, n/a-n/a (2015).

[54] Du W (2015). New asymmetric and symmetric supercapacitor cells based on nickel phosphide nanoparticles. Mater. Chem. Phys..

[55] Ramasamy K (2015). Layered ternary sulfide CuSbS_2_ nanoplates for flexible solid-state supercapacitors. J. Mater. Chem. A.

[56] Rakhi RB, Ahmed B, Hedhili MN, Anjum DH, Alshareef HN (2015). Effect of postetch annealing gas composition on the structural and electrochemical properties of Ti2CT x MXene electrodes for supercapacitor applications. Chem. Mater..

[57] Dubal DP, Suarez-Guevara J, Tonti D, Enciso E, Gomez-Romero P (2015). A high voltage solid state symmetric supercapacitor based on graphene–polyoxometalate hybrid electrodes with a hydroquinone doped hybrid gel-electrolyte. J. Mater. Chem. A.

[58] Liao Q, Li N, Jin S, Yang G, Wang C (2015). All-solid-state symmetric supercapacitor based on Co_3_O_4_ nanoparticles on vertically aligned graphene. ACS Nano.

[59] Yang Q (2017). MXene/graphene hybrid fibers for high performance flexible supercapacitors. J. Mater. Chem. A.

[60] Zhang X (2018). A flexible and high voltage symmetric supercapacitor based on hybrid configuration of cobalt hexacyanoferrate/reduced graphene oxide hydrogels. Chem. Eng. J..

[61] Mariappan VK (2019). Two dimensional famatinite sheets decorated on reduced graphene oxide: A novel electrode for high performance supercapacitors. J. Power Sources.

[62] d'Amour H, Schiferl D, Denner W, Schulz H, Holzapfel WB (1978). High-pressure single-crystal structure determinations for ruby up to 90 kbar using an automatic diffractometer. J. Appl. Phys..

[63] Santos RCR, Longhinotti E, Freire VN, Reimberg RB, Caetano EWS (2015). Elucidating the high-k insulator α-Al2O3 direct/indirect energy band gap type through density functional theory computations. Chem. Phys. Lett..

[64] Bortz ML, French RH (1989). Optical reflectivity measurements using a laser plasma light source. Appl. Phys. Lett..

[65] Yang GM, Zhang HZ, Fan XF, Zheng WT (2015). Density functional theory calculations for the quantum capacitance performance of graphene-based electrode material. J. Phys. Chem. C.

[66] Zhan C, Neal J, Wu J, Jiang D (2015). Quantum effects on the capacitance of graphene-based electrodes. J. Phys. Chem. C.

