# Supplementary material for: Experimental and computational investigation on the charge storage performance of a novel Al2O3-reduced graphene oxide hybrid electrode
Source: Sci Rep. 2023 Mar 31;13:5283. doi: 10.1038/s41598-022-23574-2 (PMC10066376; doi:10.1038/s41598-022-23574-2)
Supplement: Supplementary file 1 — Supplementary Information. [file 41598_2022_23574_MOESM1_ESM.pdf]

<u>Supplementary information</u>

Experimental and computational investigation on the charge storage performance of a novel Al_2O_3-reduced graphene oxide hybrid electrode

Satyajit Ratha[a#], Surjit Sahoo[a#], Pratap Mane[b], Balaram Polai[a], Bijoy Sathpathy[a], Brahmananda Chakraborty[c,d*], and Saroj Kumar Nayak[a*]

[a]School of Basic Sciences, Indian Institute of Technology Bhubaneswar, Argul, Khordha - 752050, India.

[b]Seismology Division, Bhabha Atomic Research Centre, Trombay, Mumbai-400085, India

[c]High Pressure & Synchroton Radiation Physics Division, Bhabha Atomic Research Centre, Trombay, Mumbai-400085, India

[d]Homi Bhabha National Institute, Mumbai-400094, India

[#] These authors have contributed equally.

*Corresponding author. Email: nayaks@iitbbs.ac.in (S.K. Nayak), brahma@barc.gov.in

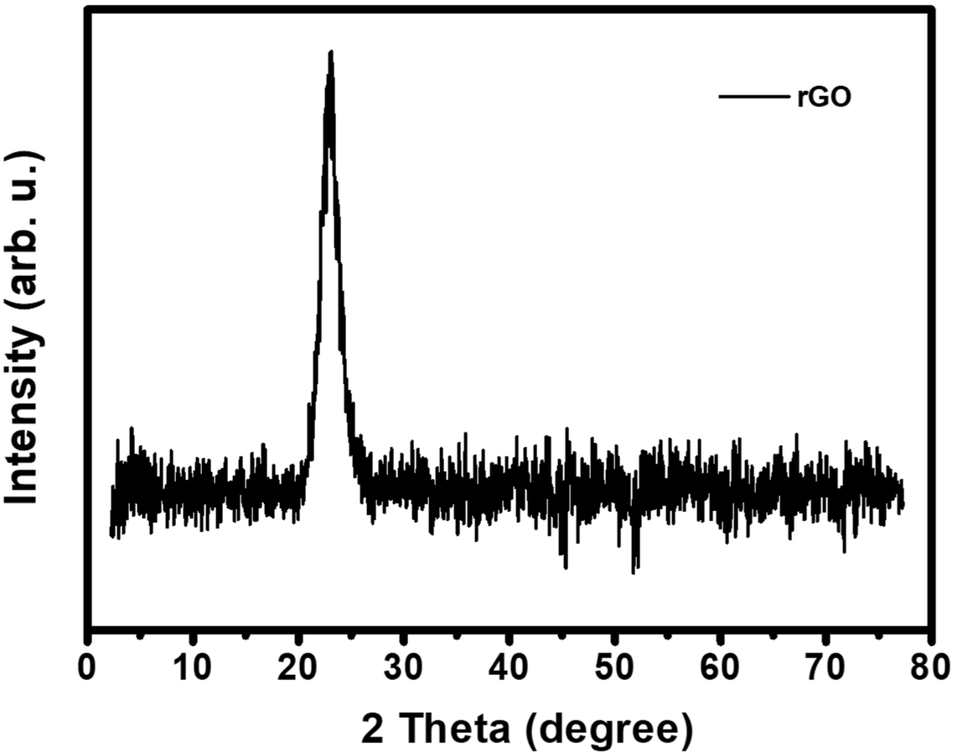

Figure S1. XRD pattern for reduced graphene oxide sample, showing a Bragg peak at ~23°. The low intensity peak is found to be subdued in the hybrid sample due to the extremely high intensity peaks of the Al_2O_3 sample.

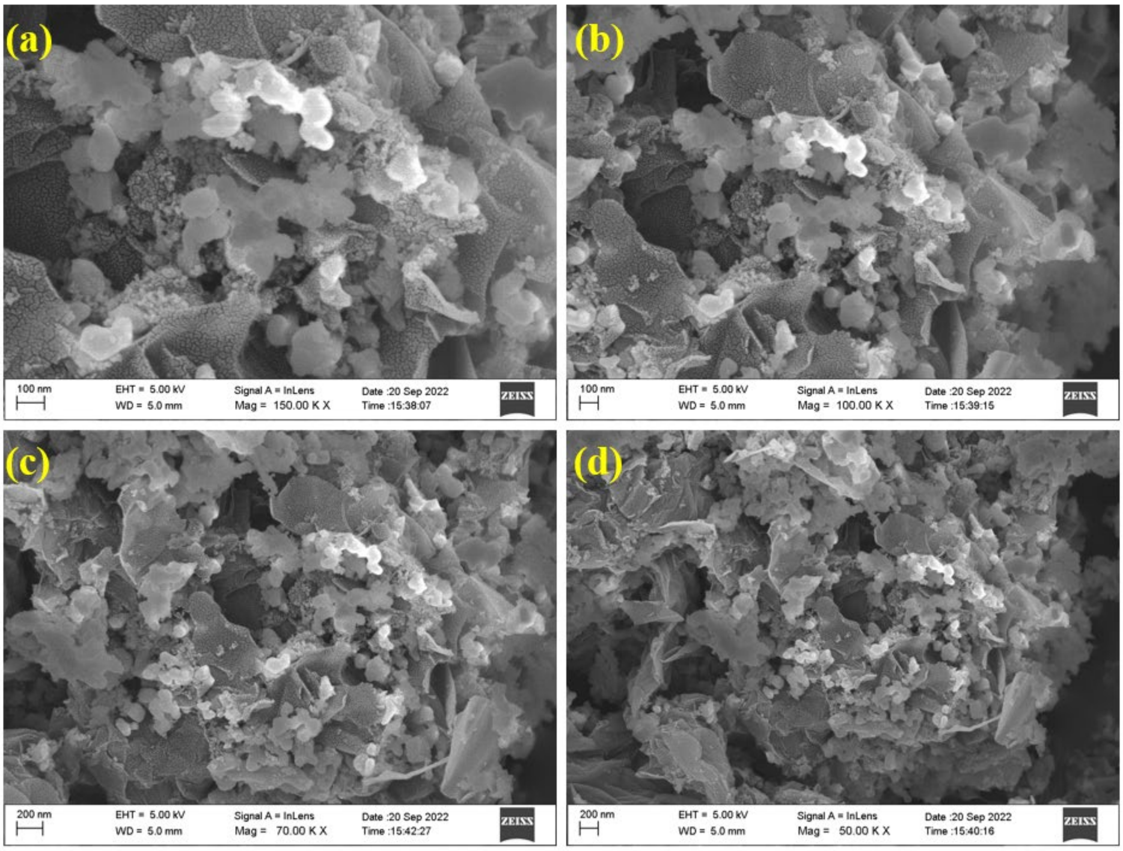

Figure S2. The SEM micrographs of the Al_2O_3-reduced graphene oxide hybrid at various magnifications, showing the incorporation of alumina nanoparticles on the reduced graphene oxide substrate.

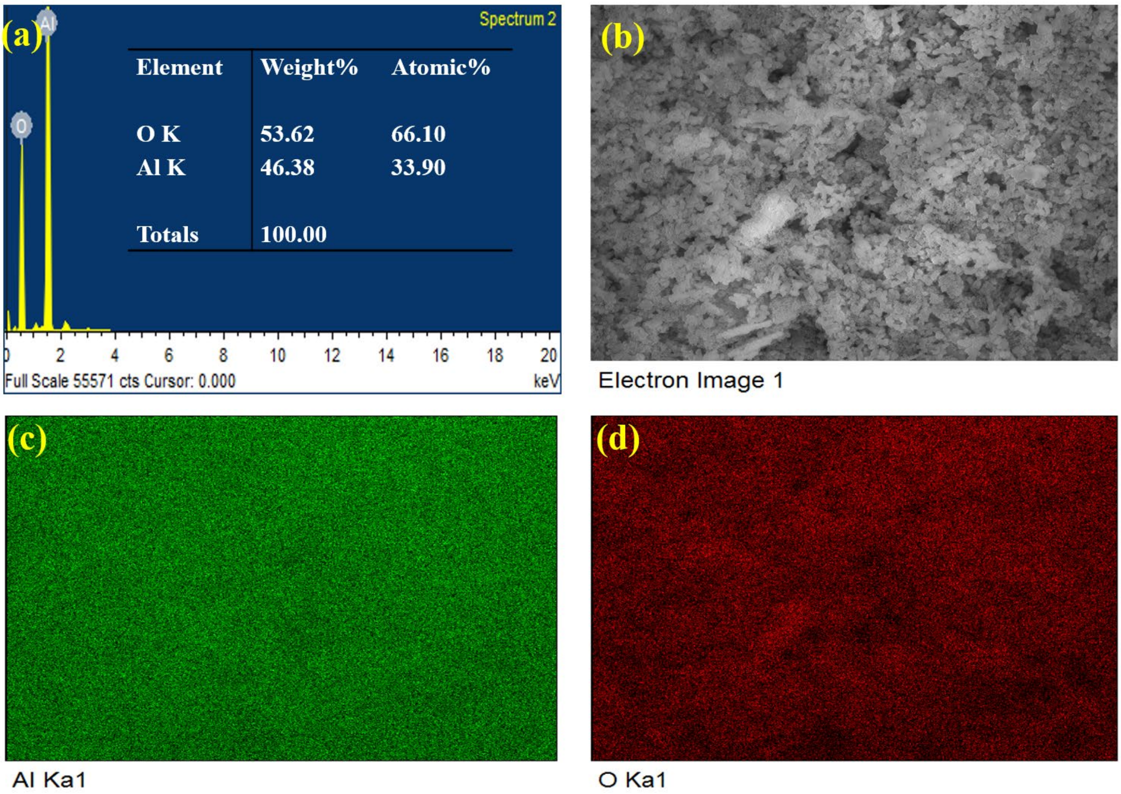

Figure S3. (a) The EDX spectrum Al_2O_3, (b) FE-SEM micrograph for elemental mapping of Al_2O_3, (c) aluminium, and (d) oxygen.

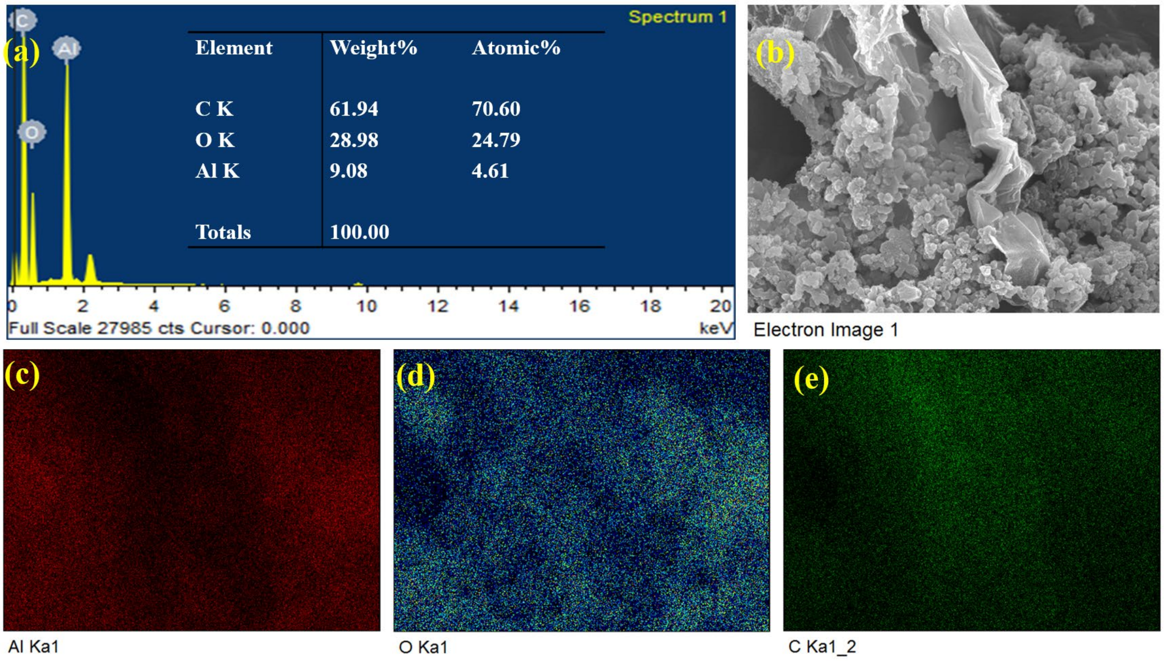

Figure S4. (a) The EDX spectrum Al_2O_3-reduced graphene oxide hybrid, (b) FE-SEM micrograph for elemental mapping of Al_2O_3-reduced graphene oxide hybrid, (c) aluminium, (d) oxygen, and (e) carbon.

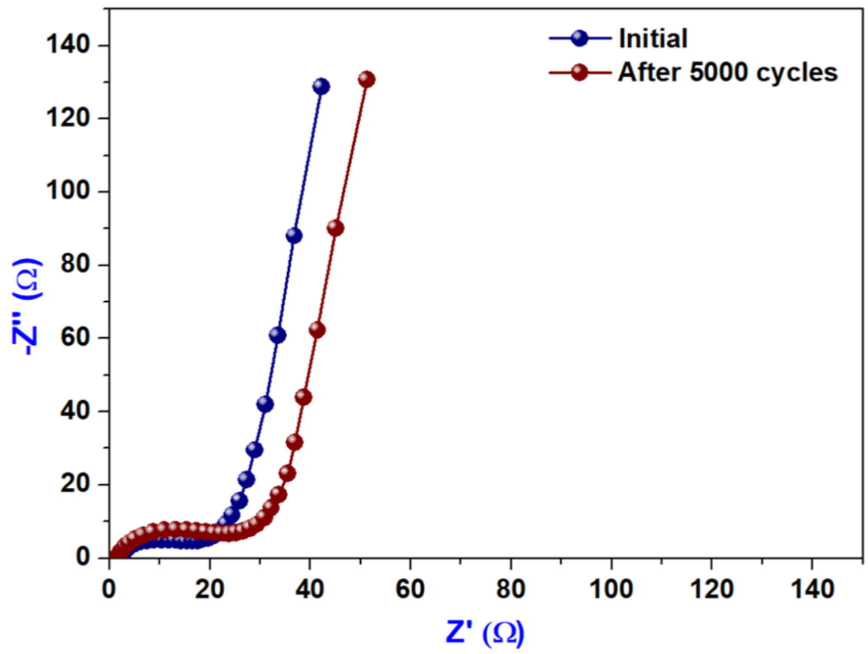

Figure S5. The comparative Nyquist plots for Al_2O_3-reduced graphene oxide hybrid electrode, obtained at initial and after cyclic stability test.

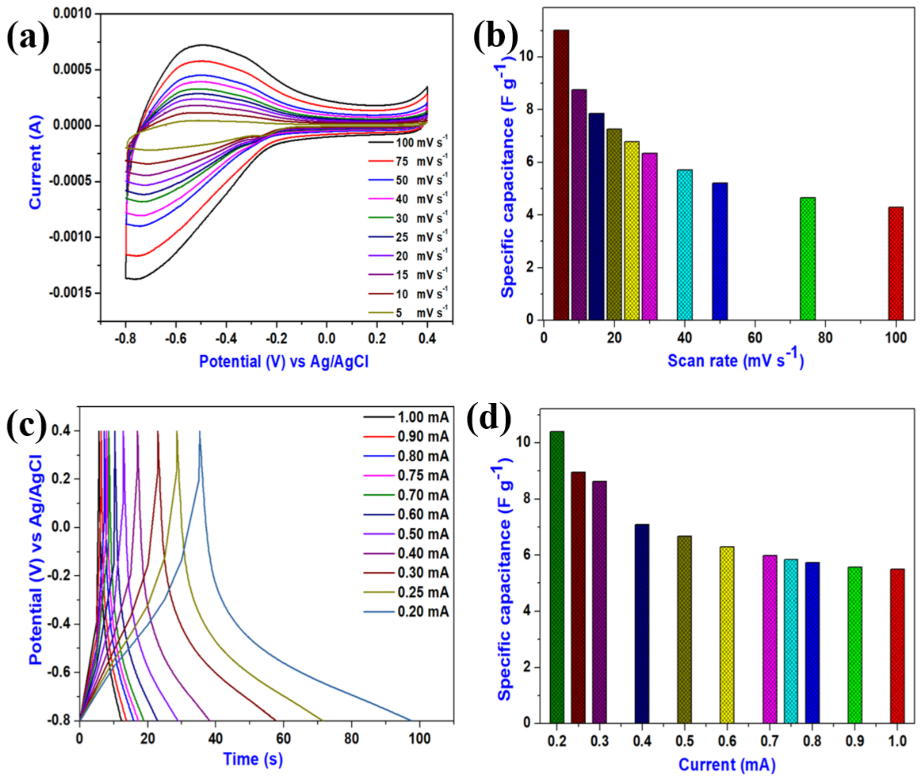

Figure S6. Electrochemical characterization of Al_2O_3 electrode. (a) Cyclic voltammetric profiles of Al_2O_3 electrode at various scan rates 5 – 100 mV s^{-1}, (b) effect of scan rates on specific capacitances of Al_2O_3 electrode, (c) charge-discharge profile of Al_2O_3 electrode at various applied currents and (d) effect of applied currents on specific capacitances of Al_2O_3 electrode.

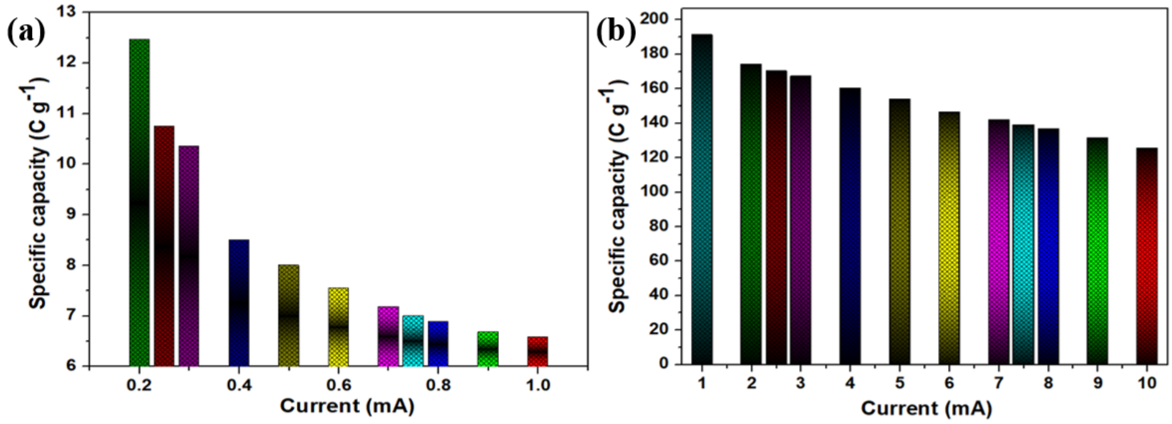

Figure S7. Effect of applied current on the specific capacity of (a) Al_2O_3 and Al_2O_3-reduced graphene oxide hybrid electrode.

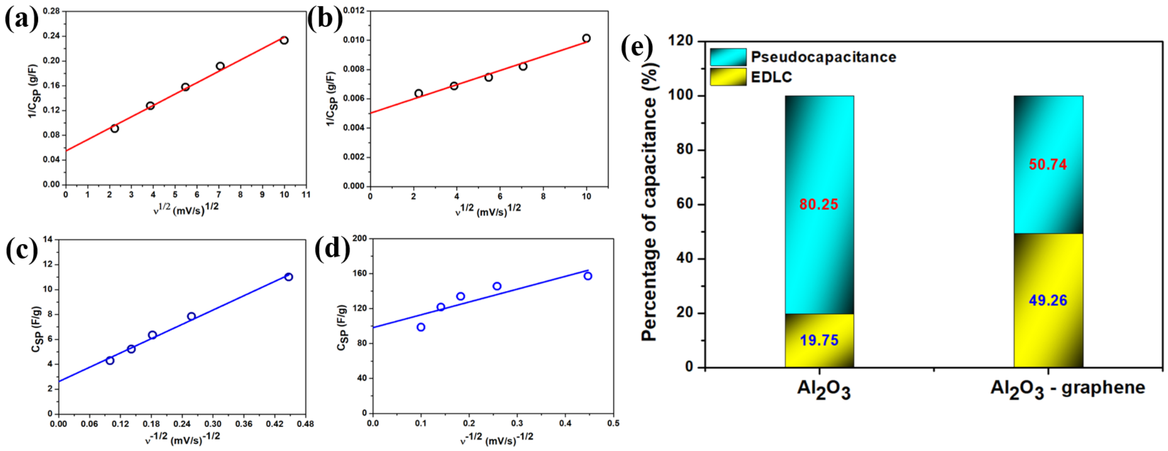

Figure S8. Trasatti plot for Al_2O_3 and Al_2O_3-reduced graphene oxide hybrid. Dependence of 1 / C_{sp} on $v^{1/2}$ for (a) Al_2O_3 electrode and (b) Al_2O_3-reduced graphene oxide hybrid electrode. Dependence of C_{sp} on $v^{-1/2}$ for (c) Al_2O_3 electrode and (d) Al_2O_3-reduced graphene oxide hybrid electrode in Na_2SO_4 electrolyte. (e) The contribution of EDLC and pseudocapacitance of Al_2O_3 electrode and Al_2O_3-reduced graphene oxide hybrid electrode.

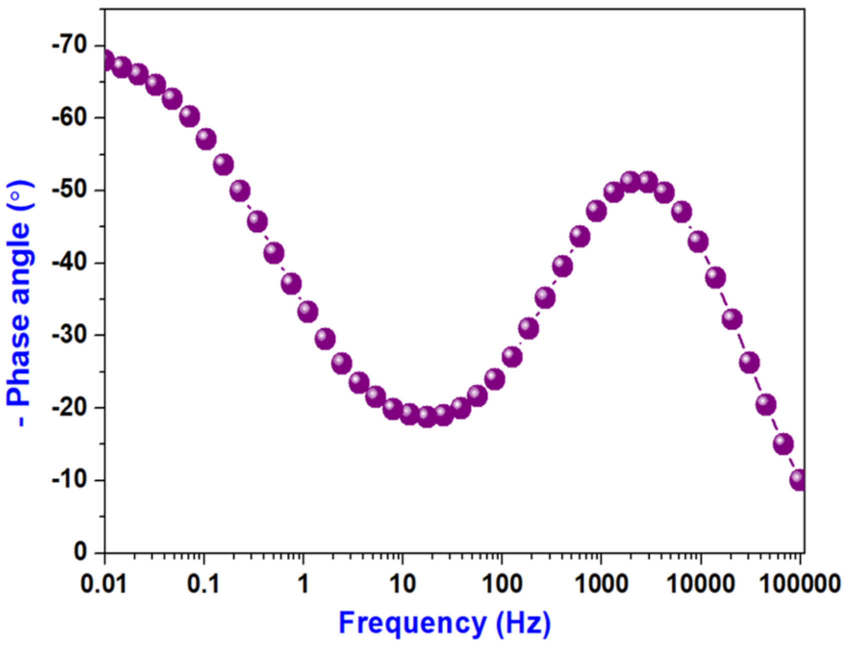

Figure S9. Bode phase angle plot of Al_2O_3-reduced graphene oxide hybrid SSD, indicating a combination of capacitive and diffusion-based charge storage process.

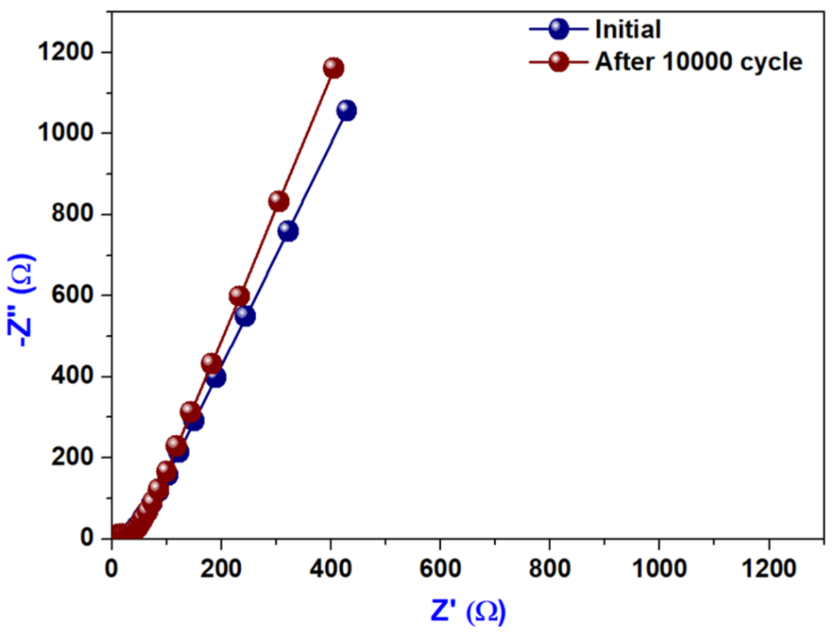

Figure S10. The comparative Nyquist plots for Al_2O_3-reduced graphene oxide hybrid SSD, taken during the initial and after the cyclic stability test.

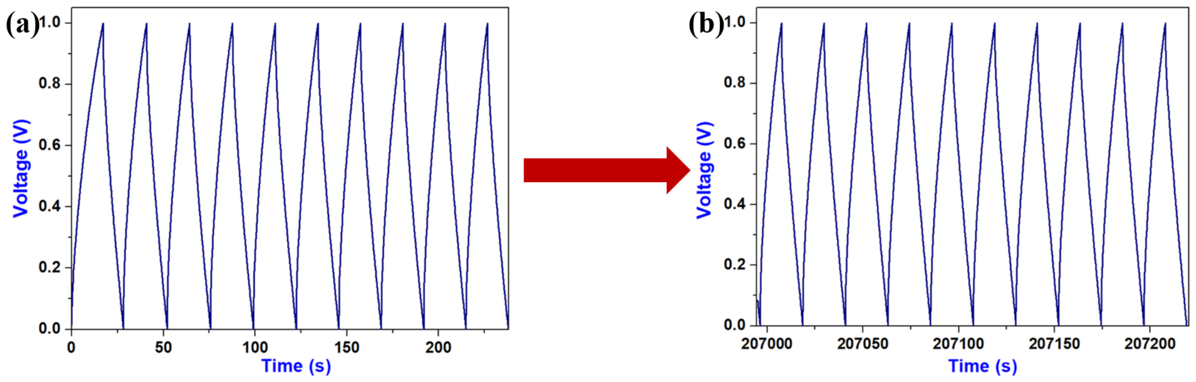

Figure S11. The initial and final ten cycles of Al_2O_3-reduced graphene oxide hybrid SSD, obtained from the long cyclic stability test.

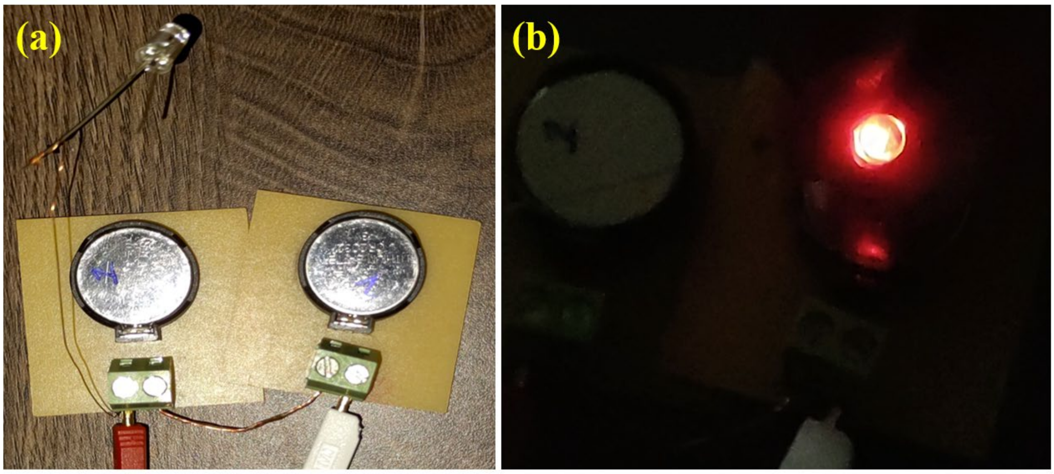

Figure S12. The practical application of Al_2O_3-reduced graphene oxide hybrid SSD coin cells (two numbers) connected in series. (a) devices in uncharged state, (b) a red LED, powered by the fully charged Al_2O_3-reduced graphene oxide hybrid SSD coin cells.

Table S1. Summary of supercapacitive performances of Al_2O_3-reduced graphene oxide hybrid electrode and reported supercapacitor electrode materials using the three-electrode system.

Material	Preparation method	Specific capacitance (F g^{-1})	Reference
$CuSbS_2$	Colloidal method	22	[1]
$CuSbSe_2$	Colloidal	48	[1]
Cu_3SbS_4	Hydrothermal	60	[2]
$FeMoO_4$ /Graphene	Hydrothermal	135	[3]
Cu_2MoS_4	Hydrothermal	135.78	[4]
ZnS	Solvothermal	32.8	[5]
WS_2	Chemical exfoliation	40	[6]
CuO	Wet chemical method	88.5	[7]
RuO_2	Chemical synthesis	50	[8]
MoO_3	Solution combustion method	108.88	[9]
MoS_2	Hydrothermal	92.85	[10]
Graphene/MnO_2	Electrochemical deposition	130	[11]
TiO_2-graphene	Sol-gel	60	[12]
Fe_3O_4-graphene	Chemical technique	65	[12]
Fe_3O_4-RGO	Electrophoretic deposition	154	[13]
ZnO-graphene	Microwave technique	109	[14]
ZnO-graphene	Solgel	95	[15]
Fe_3O_4-graphene	Hydrothermal	81	[16]
**Al_2O_3-reduced graphene oxide**	**Hydrothermal**	**157.29**	**This work**

References:

1. Ramasamy, K., Gupta, R. K., Palchoudhury, S., Ivanov, S. & Gupta, A. Layer-Structured Copper Antimony Chalcogenides ($CuSbSe_xS_{2-x}$): Stable Electrode Materials for Supercapacitors. *Chem. Mater.* **27**, 379–386 (2015).

2. Mariappan, V. K., Krishnamoorthy, K., Pazhamalai, P., Sahoo, S. & Kim, S.-J. Layered famatinite nanoplates as an advanced pseudocapacitive electrode material for supercapacitor applications. *Electrochim. Acta* **275**, (2018).

3. Wang, Y., He, P., Lei, W., Dong, F. & Zhang, T. Novel $FeMoO_4$/graphene composites based electrode materials for supercapacitors. *Compos. Sci. Technol.* **103**, 16–21 (2014).

4. Sahoo, S., Krishnamoorthy, K., Pazhamalai, P., Mariappan, V. K. & Kim, S.-J. Copper molybdenum sulfide: A novel pseudocapacitive electrode material for electrochemical energy storage device. *Int. J. Hydrogen Energy* (2018) doi:10.1016/j.ijhydene.2018.04.143.

5. Ramachandran, R. *et al.* Solvothermal synthesis of Zinc sulfide decorated Graphene (ZnS/G) nanocomposites for novel Supercapacitor electrodes. *Electrochim. Acta* **178**, 647–657 (2015).

6. Mayorga-Martinez, C. C., Ambrosi, A., Eng, A. Y. S., Sofer, Z. & Pumera, M. Transition metal dichalcogenides (MoS_2, $MoSe_2$, WS_2 and WSe_2) exfoliation technique has strong influence upon their capacitance. *Electrochem. commun.* **56**, 24–28 (2015).

7. Zhang, Y. X., Huang, M., Li, F. & Wen, Z. Q. Controlled synthesis of hierarchical CuO nanostructures for electrochemical capacitor electrodes. *Int. J. Electrochem. Sci* **8**, 8645–8661 (2013).

8. Patake, V. D. & Lokhande, C. D. Chemical synthesis of nano-porous ruthenium oxide

(RuO_2) thin films for supercapacitor application. *Appl. Surf. Sci.* **254**, 2820–2824 (2008).

9. Nagabhushana, G. P., Samrat, D. & Chandrappa, G. T. α-MoO_3 nanoparticles: solution combustion synthesis, photocatalytic and electrochemical properties. *RSC Adv.* **4**, 56784–56790 (2014).

10. Krishnamoorthy, K., Veerasubramani, G. K., Radhakrishnan, S. & Kim, S. J. Supercapacitive properties of hydrothermally synthesized sphere like MoS_2 nanostructures. *Mater. Res. Bull.* **50**, 499–502 (2014).

11. He, Y. *et al.* Freestanding three-dimensional graphene/MnO_2 composite networks as ultralight and flexible supercapacitor electrodes. *ACS Nano* **7**, 174–182 (2013).

12. Mishra, A. K. & Ramaprabhu, S. Functionalized graphene-based nanocomposites for supercapacitor application. *J. Phys. Chem. C* **115**, 14006–14013 (2011).

13. Ghasemi, S. & Ahmadi, F. Effect of surfactant on the electrochemical performance of graphene/iron oxide electrode for supercapacitor. *J. Power Sources* **289**, 129–137 (2015).

14. Ramadoss, A. & Kim, S. J. Facile preparation and electrochemical characterization of graphene/ZnO nanocomposite for supercapacitor applications. *Mater. Chem. Phys.* **140**, 405–411 (2013).

15. Bu, I. Y. Y. & Huang, R. One-pot synthesis of ZnO/reduced graphene oxide nanocomposite for supercapacitor applications. *Mater. Sci. Semicond. Process.* **31**, 131–138 (2015).

16. Song, Z. *et al.* Nano-iron oxide (Fe_2O_3)/three-dimensional graphene aerogel composite as supercapacitor electrode materials with extremely wide working potential window. *Mater. Lett.* **145**, 44–47 (2015).
